# Characterization of the TNF and IL-1 systems in human brain and blood after ischemic stroke

**DOI:** 10.1186/s40478-020-00957-y

**Published:** 2020-06-05

**Authors:** Bettina H. Clausen, Martin Wirenfeldt, Sofie S. Høgedal, Lars H. Frich, Helle H. Nielsen, Henrik D. Schrøder, Kamilla Østergaard, Bente Finsen, Bjarne W. Kristensen, Kate L. Lambertsen

**Affiliations:** 1 Department of Neurobiology Research, Institute of Molecular Medicine, University of Southern Denmark, J.B. Winsloewsvej 21, st, DK-5000 Odense C, Denmark; 2 BRIDGE, Inter-Disciplinary Guided Excellence, Department of Clinical Research, University of Southern Denmark, DK-5000 Odense C, Denmark; 3 Department of Pathology, Odense University Hospital, Odense, J.B. Winsloewsvej 15, DK-5000 Odense C, Denmark; 4 Department of Neurology, Odense University Hospital, J.B. Winsloewsvej 4, DK-5000 Odense C, Denmark; 5 Orthopedic Research Unit, University of Southern Denmark, DK-5000 Odense C, Denmark; 6 OPEN, Open Patient data Explorative Network, Odense University Hospital, Department of Clinical Research, University of Southern Denmark, J.B. Winsloewsvej 9a, DK-5000 Odense, Denmark

**Keywords:** Apoplexy, Microglia, Leukocytes, Cytokines

## Abstract

Preclinical and clinical proof-of-concept studies have suggested the effectiveness of pharmacological modulation of inflammatory cytokines in ischemic stroke. Experimental evidence shows that targeting tumor necrosis factor (TNF) and interleukin (IL)-1 holds promise, and these cytokines are considered prime targets in the development of new stroke therapies. So far, however, information on the cellular expression of TNF and IL-1 in the human ischemic brain is sparse.

We studied 14 cases of human post-mortem ischemic stroke, representing 21 specimens of infarcts aged 1 to > 8 days. We characterized glial and leukocyte reactions in the infarct/peri-infarct (I/PI) and normal-appearing tissue (NAT) and the cellular location of TNF, TNF receptor (TNFR)1 and TNFR2, IL-1α, IL-1β, and IL-1 receptor antagonist (IL-1Ra). The immunohistochemically stained tissue sections received a score reflecting the number of immunoreactive cells and the intensity of the immunoreactivity (IR) in individual cells where 0 = no immunoreactive cells, 1 = many intermediately to strongly immunoreactive cells, and 2 = numerous and intensively immunoreactive cells. Additionally, we measured blood TNF, TNFR, and IL-1 levels in surviving ischemic stroke patients within the first 8 h and again at 72 h after symptom onset and compared levels to healthy controls.

We observed IL-1α and IL-1β IR in neurons, glia, and macrophages in all specimens. IL-1Ra IR was found in glia, in addition to macrophages. TNF IR was initially found in neurons located in I/PI and NAT but increased in glia in older infarcts. TNF IR increased in macrophages in all specimens. TNFR1 IR was found in neurons and glia and macrophages, while TNFR2 was expressed only by glia in I/PI and NAT, and by macrophages in I/PI. Our results suggest that TNF and IL-1 are expressed by subsets of cells and that TNFR2 is expressed in areas with increased astrocytic reactivity. In ischemic stroke patients, we demonstrate that plasma TNFR1 and TNFR2 levels increased in the acute phase after symptom onset compared to healthy controls, whereas TNF, IL-1α, IL-1β, and IL-1Ra did not change.

Our findings of increased brain cytokines and plasma TNFR1 and TNFR2 support the hypothesis that targeting post-stroke inflammation could be a promising add-on therapy in ischemic stroke patients.

## Introduction

Inflammation is integral to the pathophysiology of ischemic stroke and a prime target for the development of new stroke therapies. The inflammatory cytokines interleukin (IL)-1 and tumor necrosis factor (TNF) are pivotal in regulating immune responses following ischemic stroke (reviewed in [31]) and are potential targets in stroke therapy. IL-1 comprises two agonists, IL-1α and IL-1β, both signaling through IL-1 receptor type I that is expressed by neurons, glia, and endothelial cells [1, 36]. In response to stroke, IL-1β is rapidly upregulated in the blood [41, 55, 69] and brain [45] and exacerbates injury in experimental animal models (reviewed in [31]). The IL-1 receptor antagonist (IL-1Ra) is a natural competitive inhibitor of IL-1 signaling [23] and modulates the size of ischemic infarcts in experimental animal models [12, 37, 44, 49]. An IL-1β haplotype in postmenopausal women and hypertensive persons [3], and an IL-1α haplotype in Koreans [64] are associated with increased stroke risk.

TNF is an inflammatory cytokine with both beneficial and detrimental properties for the CNS (reviewed in [28, 31]). It is expressed at constitutively low levels in the healthy CNS and, along with its two known receptors TNFR1 and TNFR2, increases after both experimental and human ischemic stroke [10, 17, 30, 45, 52, 61]. Plasma TNF levels are increased in stroke patients compared with controls [5], and common polymorphisms of the TNF gene promoter leading to increased circulating TNF levels appear to be associated with cardiovascular risk factors and ischemic stroke in Asians [14, 63]. TNF exists as both membrane-bound TNF (mTNF) and soluble TNF (solTNF), and emerging experimental data suggest that solTNF is detrimental whereas mTNF has protective properties and is necessary for the maintenance of innate immunity [6, 35, 38, 47, 58, 70].

The brain is extremely susceptible to both extrinsic and intrinsic challenges, and ischemic stroke initiates an acute and long-lasting inflammatory response characterized by glial activation and leukocyte infiltration. The inflammatory response is accompanied by increased cytokine production, both centrally in the brain but also in the periphery (reviewed in [7, 28]). In the present study, we determined the cellular expression of IL-1α, IL-1β, IL-1Ra, TNF, TNFR1, and TNFR2 in post-mortem ischemic brain tissue and, in parallel, the expression of the microglial/macrophage markers CD68, CD45 and Iba1 and the astrocytic marker glial fibrillary acidic protein (GFAP). In addition, we compared blood levels of IL-1α, IL-1β, IL-1Ra, TNF, TNFR1, and TNFR2 in samples from ischemic stroke survivors and healthy controls.

## Materials and methods

### Participants and recruitment

The prospective part of this study was conducted at the Department of Neurology and the Department of Orthopaedic Surgery, Odense University Hospital (OUH), from September 2017 to April 2018 and from May 2016 to February 2019, respectively.

Neurological patients presenting with classic clinical symptoms of stroke were eligible if they were over 18 years old and admitted within 48 h of symptom onset (*n* = 34). Both men and women were included, as were patients who underwent treatment with thrombolysis or thrombectomy. Exclusion criteria were the presence of a space-occupying lesion, sinus thrombosis, pregnancy, and the inability to write and understand Danish. A standard non-contrast head CT scan was taken at admission on all patients suspected of stroke. Some had MRI taken for further diagnostic clarification. All scans were assessed by radiologists at OUH for ischemic or hemorrhagic changes in the neural tissue. Patients were diagnosed by a combination of clinical history, physical examination, and CT scans, and only patients diagnosed with ischemic stroke were included. Patients received treatment according to the national standards in Denmark.

Controls (*n* = 7) were recruited through the relatives. Further, orthopedic patients over 18 years old undergoing surgery for rotator cuff tendon tear (*n* = 21) were included. Exclusion criteria included diabetes, autoimmune diseases, previous shoulder surgery, a fractured or dislocated shoulder, and the inability to write and understand Danish.

### Outcome measures

Stroke patients were assessed on admission by a clinician using the Scandinavian Stroke Scale (SSS), which measures stroke severity based on physical examination on a scale from 0 to 58, with milder symptoms giving a higher score [22]. Modified Rankin Scale (mRS), a 7-step scale from 0 (no functional disabilities) to 6 (death), was used to assess functional outcome 3 months after discharge via a telephone interview.

### Other data collection

Data were collected on age, sex, weight, height, smoking and drinking habits, pre-existing diabetes, and use of anti-inflammatory medication. In ischemic stroke patients, we further collected data on first time of clinical symptoms, symptoms at time of admission, and type and location of the lesion. In orthopedic patients, we further collected data on pre-existing rheumatic disease. None displayed any signs of rheumatic disease. As our healthy controls and orthopedic patients were comparable in sex, age, body mass index (BMI), drinking habits, and anti-inflammatory medication, although not smoking habits (Suppl. Table 1), we combined them into one control group for further analysis.

### Procedures

Venous blood was drawn in neurological patients at admission (*n* = 34) and 72 h later (non-discharged patients, *n* = 9) and in orthopedic patients just prior to surgery for rotator cuff tendon tear (*n* = 21, only plasma). Blood was collected in 4 ml vacutainer and EDTA tubes and then centrifuged. Samples were aliquoted and kept at − 80 °C until analysis. Differential leukocyte counts (total leukocyte, neutrophil, lymphocyte, and monocyte counts) in orthopedic patients were all within normal range (Suppl. Table 1).

### Cytokine and cytokine receptor analysis

TNF, TNFR1, TNFR2 concentrations in plasma, and IL-1α, IL-1β, and IL-1Ra concentrations in serum and plasma were analyzed in duplicate using commercially available V-Plex Plus human kits (Mesoscale, Rockville, USA) according to the manufacturer’s instructions. Prior to measurement, the samples were diluted two-fold in Diluent 41. MSD Discovery Workbench software was used for analysis [8]. Samples with a coefficient of variation (CV) values > 25% in individual analyses were excluded. Only samples without repeated freeze-thaw cycles were used.

### Post-mortem brain tissue

The retrospective part of this study included 21 autopsy specimens from 14 patients admitted to OUH in 2000–2005. These were nine males (median age 61 years, interquartile range (IQR) 38–76 years) and five females (median age 78 years, IQR 67–83 years). Table 1 shows age, sex, infarcted brain area, age of infarct, and cause of death. Mixed organ tissue blocks were used as controls for immunohistochemical staining, as was brain tissue free of brain-specific disease (*n* = 3). Parallel tissue sections were used in previous studies [10, 12, 31, 32, 46].

**Table 1:** Clinical data from *post-mortem* brain tissue

Case	Sex/Age at death	Infarcted brain area	Infarct age	Cause of death
1	M/76	Left temporal lobe	< 7 days	Multiorgan failure due to sepsis
2	F/73	Right temporal lobe	< 7 days	Rupture of thoracic aortic aneurysm
3	F/78	Right hemisphere	2 days	Perforated gastric ulcer with peritonitis and infarct
4	M/67	Left temporal lobe	< 7 days	Cardiac arrest
5	M/61	Right parietal lobe	< 1 day > 7 days	Multiorgan failure due to sepsis, cardiac infarct and endocarditis
6	F/83	Right hippocampus Cerebellum	1 day > 7 days	Pulmonary embolism
7	F/80	Right frontal lobe Medulla Pons Striatum	1 day < 5 days 1 day < 7 days	Bronchopneumonia
8	M/68	Left parietal lobe	< 1 day	Cerebral infarct, pneumonia
9	M/68	Caudate nucleus Insula	> 7 days > 7 days	Bronchopneumonia
10	M/59	Right parietal lobe	> 7 days	Pneumonia
11	M/57	Left internal capsule	> 7 days	Pneumonia
12	F/67	Right occipital lobe Right occipital lobe	1 day > 2 days	Pulmonary infarcts Respiratory distress syndrome
13	M/38	Right parietal lobe	< 5 days	Cerebral herniation, subdural empyema, ARDS
14	M/48	Right temporal lobe	> 7 days	Pneumonia, hypoglycemia

*ARDS* acute respiratory distress syndrome, *F* female, *M* male

### Preparation of tissue

Human post-mortem tissue encompassing infarcted brain tissue was formalin-fixed, embedded in paraffin, and cut into 2 μm thick, serial sections on a microtome. Tissue sections were then dewaxed in xylene and rehydrated in ethanol. For immunohistochemical staining, endogenous peroxidase activity was quenched using 1.5% hydrogen peroxide in Tris-buffered saline (TBS). For optimal staining protocols, heat-induced epitope retrieval was performed using T-EG buffer (10 mM Tris, 0.5 mM EGTA, pH 9) for chromogen staining and citrate buffer (10 mM citrate, pH 6) for fluorescence staining.

### Hematoxylin and eosin (HE) staining

For visualization of nuclei and cytoplasmic inclusions, one section from each specimen was stained using HE according to standard protocols at the Department of Pathology, OUH. HE-stained tissue sections were evaluated by two independent neuropathologists.

### Immunohistochemistry

Immunohistochemical staining was performed using the Dako autostainer platform (Dako, Denmark) as previously described [24]. Sections were stained using the following primary antibodies: mouse anti-CD68 (clone PG-M1, 1:100, Dako), mouse anti-CD45 (clone 2B11, 1:200, Dako), rabbit anti-Iba1 (ionized calcium binding adaptor molecule 1, 1:1000, Wako), rabbit anti-GFAP (1:2000, Dako), mouse anti-neurofilament (NF) (phosphorylated and non-phosphorylated NF-heavy chain; clone N52, 1:1000, Sigma-Aldrich), mouse anti-IL-1α (clone 4414, 1:1200, R&D Systems), mouse anti-IL-1β (clone 2E8, 1:50, BioRad), rabbit anti-TNF (1:100, ThermoFisher Scientific), rabbit anti-TNFR1 (clone H-271, 1:50, Santa Cruz), rabbit anti-TNFR2 (1:50, Sigma-Aldrich), and rat anti-IL-1Ra (clone 40,007, 1:1500, R&D Systems). The antigen-antibody complex was visualized using EnVision+System horse-radish peroxidase-labelled Polymer (Dako), PowerVision+Poly-HRP IHC (AH Diagnostics), or CSAII (Dako) detection systems.

### Control reactions

Controls for antibody specificity and non-specific staining were performed by substituting the primary antibodies with rabbit IgG (TNF, TNFR1, and TNFR2), mouse IgG_2a_ (IL-1α), mouse IgG1 (IL-1β), or rat IgG_2a_ (IL-1Ra) in the same IgG concentrations or by omitting the primary antibody in the protocol. Immunoabsorption was performed using a mixture of the primary antibody and a 100-fold excess of a recombinant human (rh) protein (rhTNF (210-TA); rhTNFR1/TNFRSF1A (636-R1); rhTNFR2/TNFRSF1B (aa 24–206; all from R&D Systems); and rhIL-1β (rcyec-hil1b, InvivoGen)). Controls were devoid of staining or showed a reduced signal (Suppl. Fig. 1).

### Immunofluorescent and immunohistochemical double staining

Sections were bleached in Autofluorescence Eliminator Reagent (Millipore) according to the manufacturer’s guidelines. This treatment completely removed autofluorescence in the tissue (Suppl. Fig. 2). Sections were then pre-incubated with 5% normal serum from secondary antibody species diluted in phosphate-buffered saline (PBS) containing 0.25% Triton (PBS-T). Sections were incubated overnight with primary antibodies diluted in PBS-T as described above for IL-1α, IL-1β, TNF, TNFR1, and TNFR2. Next day, sections were rinsed 2 × 5 min in PBS, followed by 3 × 10 min in PBS-T, and incubated overnight with specific antibodies directed against Iba1 (1:600), NeuN-488 (clone EPR12763, 1:250, Abcam), CD68-FITC (clone KP1, 1:500, Dako), GFAP-Cy3 (clone G-A-5, 1:500, Sigma-Aldrich), or NF-heavy chain (clone TA51, 1:50, Millipore). The following day, sections were rinsed in PBS-T for 10 min and incubated with Alexa-488-conjugated donkey anti-rat, Alexa-488-conjugated goat anti-rabbit, Alexa-488-conjugated donkey anti-rabbit, Alexa-555-conjugated donkey anti-mouse, or Alexa-594-conjugated donkey anti-rabbit (all 1:500 and from Invitrogen) for 2 h at room temperature. Finally, sections were rinsed 3x in PBS and 1x in TBS before being mounted in ProLong Gold Antifade Reagent with 4′,6-diamidine-2′-phenylindole dihydrochloride (DAPI) (Thermo Fisher Scientific). Immunohistochemical double staining for IL-1Ra and CD45 was performed as previously described [12].

### Scoring of cell-specific staining intensity

**Fig. 1:**
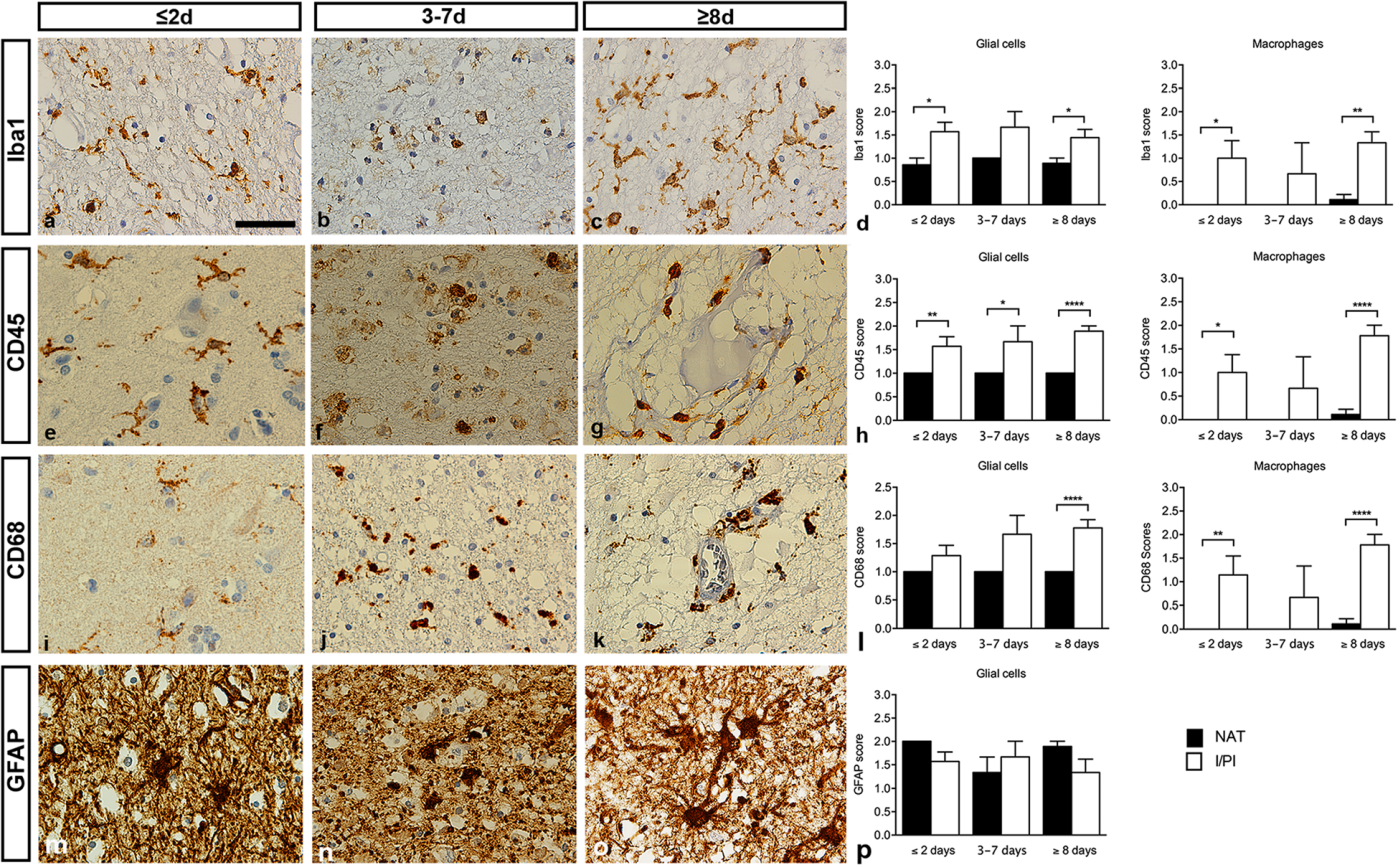
Characterization of glial reactivity in post-mortem ischemic stroke brain tissue. **a-c** Iba1 staining demonstrating ramified microglia and round macrophage-like cells in ≤2-day-old (**a**), 3–7-day-old (**b**), and ≥ 8-day-old (**c**) infarcts. **d** Scoring of Iba1 staining intensity demonstrating higher Iba1 expression in I/PI than in NAT in both microglia and macrophages. **e-h** CD45 staining demonstrating ramified microglia in ≤2-day-old infarcts (**e**) and round macrophage-like cells in 3–7-day-old (**f**) and ≥ 8-day-old (**g**) infarcts. **h** Scoring of CD45 staining intensity demonstrating higher CD45 expression in I/PI than in NAT in both microglia and macrophages. CD45^+^ macrophages were rarely detected in NAT. **i-k** CD68 staining demonstrating ramified microglia in ≤2-day-old infarcts (**i**) and round macrophage-like cells in 3–7-day-old (**j**), and ≥ 8-day-old (**k**) infarcts. (**l**) Scoring of CD68 staining intensity demonstrating higher CD68 expression in I/PI than in NAT in both microglia and macrophages. CD68^+^ macrophages were rarely detected in NAT. **m-o** GFAP staining demonstrating gemistocytic astrocytes in a ≤ 2-day-old infarct (**m**), shrunken astrocytes with absence of astrocytic processes in a 3–7-day-old infarct (**n**), and gemistocytic astrocytes in a ≥ 8-day-old infarct (**o**). **p** Scoring of GFAP staining intensity demonstrating comparable staining intensity in I/PI and NAT at all timepoints. GFAP, glial fibrillary acidic protein; Iba1, ionized calcium-binding adapter molecule 1; I/PI, infarct/peri-infarct; NAT, normal appearing tissue. Results are presented as mean ± SD. *p < 0.05, **p < 0.01, ***p < 0.001; one-way ANOVA followed by Sidak’s multiple comparisons test (*n* = 3–9/group). Scale bar = 40 μm

The sections were scored independently by a neuropathologist and a neurobiologist who were blinded to the age and the location of infarct, and cause of death. Three categories were used to generate a score reflecting the number of immunoreactive cells and the intensity of the immunoreactivity (IR) of individual cells: 0 = no immunoreactive cells, 1 = many intermediately to strongly immunoreactive cells, 2 = numerous and intensively immunoreactive cells [12, 43] (for representative images of the scoring system, please refer to Suppl. Fig. 3). A low sensitivity 0–2 scoring scale was applied because of the multiple variations in post-mortem autolysis and fixation time this autopsy tissue has been exposed to, and which could not be controlled for prior to the actual immunohistochemical staining. Variation in post-mortem autolysis and fixation time could have more impact on the data and introduce bias in the results with the use of more precise quantification methods, such as computerized pixel count. Based on our previous findings of a microglial/macrophage expression of TNF, TNFR2, IL-1α, IL-1β, and IL-1Ra in experimental stroke models [12, 29, 30], cells of particular interest were glia and infiltrating macrophages, although neuronal expression was also evaluated. Cells were scored based on their morphology. Microglia were characterized by a ramified morphology (representative images are shown in Fig. 1a,e,i) and macrophages by a round morphology (representative images are shown in Fig. 1g,k). The intensity was evaluated in normal-appearing tissue (NAT) and in infarct/peri-infarct (I/PI) tissue. Scoring was performed using a conventional Leica bright field microscope.

### Statistical analysis

To compare stroke patients and controls, we used chi-square test for smoking, alcohol, and anti-inflammatory medication, Fisher’s exact test for sex, and Mann-Whitney test for age and BMI. For analysis of blood markers, the Shapiro-Wilk normality test was used to test for normal distribution of data, ROUT’s test to detect outliers, and Kruskal-Wallis test followed by Dunn’s multiple comparison to compare groups. One-way ANOVA test followed by Sidak’s multiple comparison was used to compare NAT and I/PI tissue.

Data are presented as mean ± SD or as median with IQR [25;75], and *p* ≤ 0.05 was considered statistically significant. Statistical analyses were performed using the Prism 5 software for Mac (GraphPad).

## Results

### Glial and macrophage reactivity in post-mortem ischemic brain tissue

We scored glial and macrophage reactivity in parallel I/PI and NAT brain tissue sections using the microglial/macrophage markers Iba1 (Fig. 1a-d), CD45 (Fig. 1e-h), and CD68 (Fig. 1i-l), and the astrocytic marker GFAP (Fig. 1m-p) in ≤2-day-old, 3–7-day-old, and ≥ 8-day-old infarcts (Fig. 1d,h,l,p). At all timepoints, intensely Iba1 immunoreactive (Iba1^+^) microglia and macrophages were present in I/PI and to a lesser extent Iba1^+^ microglia in NAT (Fig. 1d). In ≤2-day-old and ≥ 8-day-old infarcts, the Iba1 score was significantly higher in I/PI than in NAT for both microglia and macrophages. The Iba1 score for macrophages was very low in NAT (Fig. 1d, right graph). Similar findings were observed for CD45 (Fig. 1h) and CD68 (Fig. 1k), despite a tendency to increased IR in I/PI over time.

In ≤2-day-old lesions, microglial CD68 IR and infiltration of CD68^+^ macrophages were minimal. In one cortical infarct (Case #3, Table 1), a few CD68^+^ macrophages appeared to have infiltrated from meningeal vessels into the cortical layer, and CD68^+^ perivascular cells were present. Microglial CD68 IR, however, was minimal. At this early stage, subtle microglial reactivity and macrophage infiltration were observed in several cases. One case of infarction in the pyramids in the medulla oblongata (Case #7, Table 1), however, displayed pronounced infiltration of CD68^+^ macrophages in a pattern almost encircling both pyramids. In another case with infiltration of CD68^+^ meningeal and perivascular macrophages (Case #12, Table 1), parenchymal microglial CD68 IR was sparse.

In up to 7-day-old lesions, CD68^+^ microglia and infiltration of CD68^+^ macrophages were observed in the peri-infarct, while the infarcted area per se appeared less infiltrated by macrophages at this early stage. In one infarct, affecting the striatum and internal capsule (Case #11, Table 1), CD68^+^ microglia were located among the myelinated axons as an indication of Wallerian degeneration. Infarcts up to 7-days-old had pronounced infiltration of CD68^+^ macrophages, and microglial CD68 IR concomitantly increased in peri-infarct areas. In one case (Case #10, Table 1), CD68^+^ microglial rod cells appeared in the cerebral cortex adjacent to the infarct.

In older infarcts, CD68^+^ microglia were observed at the border of the infarct, but most microglial activity had subsided. CD68^+^ macrophages were present in the parenchyma particularly perivascularly. One old cortical infarct with laminar necrosis (Case #4, Table 1) showed extensive CD68^+^ macrophage infiltration in the laminar infarct and pronounced adjacent microglial reactivity.

The score for Iba1 generally paralleled the score for CD68 but showed different intensity. As microglia typically labeled more intensely with the anti-Iba1 antibody than with the anti-CD68 antibody, the ramified morphology of microglia was most clearly visible in the Iba1 staining. In contrast, macrophages labeled more intensely when stained for CD68. In the older infarcts, microglia generally displayed a ramified morphology though microglial Iba1 IR was still enhanced.

Regarding the score for CD45 in early infarcts, CD45^+^ microglia occasionally appeared intensified around lesions. In 3–7-day-old infarcts and in older lesions, microglial CD45 IR was less pronounced in peri-infarct areas than in the corresponding Iba1 staining, consistent with the CD45^low^ phenotype of microglia. CD45^+^ macrophages were not as intensely labeled as when stained for CD68.

Similar to previous findings [25, 33], the GFAP score, reflecting the number of GFAP^+^ astrocytes and the GFAP IR of individual astrocytes, was decreased within the infarct, creating an outline of the infarcted area compared to the surrounding GFAP^+^ vital peri-infarct (Fig. 1p). At all timepoints investigated, GFAP IR was enhanced in the cortical peri-infarct areas compared to the infarct.

### IL-1β IR is present in neurons, glia, and infiltrating macrophages

**Fig. 2:**
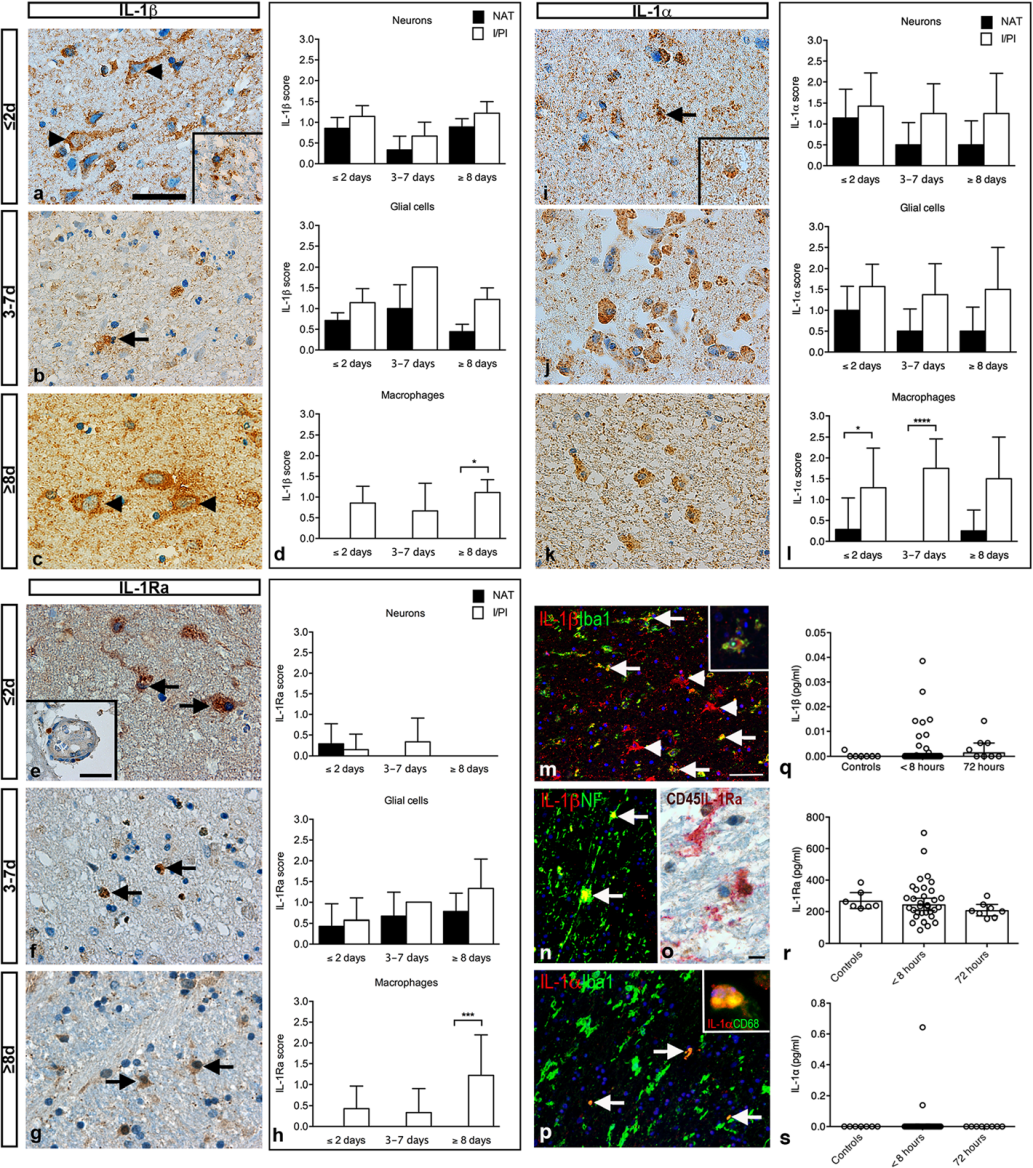
Characterization of cellular IL-1 expression and serum IL-1 levels in ischemic stroke. **a** IL-1β immunohistochemical staining demonstrating IL-1β^+^ neurons (arrowheads) and IL-1β^+^ glial cells (insert) in a ≤ 2-day-old infarct. **b** IL-1β^+^ glial cell (arrow) located in a 3–7-day-old infarct. **c** IL-1β^+^ neurons (arrowheads) in a ≥ 8-day-old infarct. **d** Scoring of IL-1β staining intensity showing comparable IL-1β expression in neurons and glial cells in I/PI and NAT, whereas IL-1β was only expressed in macrophages located in I/PI. **e** IL-1Ra immunohistochemical staining demonstrating IL-1Ra^+^ glial cells (arrows) and IL-1Ra^+^ macrophages extravasating from capillaries (insert). **f** IL-1Ra^+^ macrophages (arrows) in I/PI in a 3–7-day-old infarct. **g** IL-1Ra^+^ glia (arrows) in a ≥ 8-day-old infarct. **h** Scoring of staining intensity showed that IL-1Ra expression was low in neurons in I/PI and NAT early after stroke and was absent in older infarcts. IL-1Ra was mainly expressed in glia located in NAT and I/PI, and in IL-1Ra^+^ macrophages located in I/PI. IL-1Ra^+^ macrophages were absent from NAT. **i** IL-1α immunohistochemical staining demonstrating IL-1α^+^ glial cell (arrow) and IL-1α^+^ macrophage (insert) in a ≤ 2-day-old infarct. **j** IL-1α^+^ macrophages in a 3–7-day-old infarct. **k** IL-1α^+^ macrophages in a ≥ 8-day-old infarct. **l** Scoring of IL-1α staining intensity showing comparable IL-1α expression in neurons and glia in I/PI and NAT, whereas IL-1α was primarily expressed in macrophages in I/PI and to a lesser extent in macrophages in NAT. **m** Immunofluorescent staining demonstrating co-localization of IL-1β (red) with Iba1^+^ (green) microglia (arrows) and Iba1^+^ (green) macrophages (insert). Note that not all IL-1β^+^ cells (arrowheads) co-localized to Iba1^+^ cells. **n** Immunofluorescent staining demonstrating co-localization of IL-1β (red) with NF^+^ (green) neurons (arrows). **o** Immunohistochemical double staining demonstrating co-localization of IL-1Ra^+^ (red) and CD45^+^ (brown) microglia. **p** Immunofluorescent staining demonstrating co-localization of IL-1α (red) with Iba1^+^ (green) microglia (arrows) and CD68^+^ (green) macrophages (insert). **q-s** V-Plex analysis demonstrating comparable plasma levels of IL-1β (**q**), IL-1Ra (**r**), and IL-1α (**s**) between controls (*n* = 7) and ischemic stroke patients at < 8 h (*n* = 34) and 72 h (*n* = 9) after symptom onset. For IL-1β, CV > 25% in one 72-h sample, for IL-1RA CV > 25% in one < 8-h sample and in one 72-h sample. IL-1α, interleukin-1alpha; IL-1β, interleukin-1beta; IL-1Ra, interleukin-1 receptor antagonist; I/PI, infarct/peri-infarct; NF, neurofilament; NAT, normal appearing tissue. Results are presented as mean ± SD (staining intensity) or mean with IQR (V-plex analysis). *p < 0.05, **p < 0.01, ***p < 0.001; one-way ANOVA followed by Sidak’s multiple comparisons test (scoring of staining intensity, *n* = 3–9/group) or Kruskal Wallis test followed by Dunn’s multiple comparison test (V-plex analysis). Scale bars: a-c, e-g, i-k = 40 μm, m-n, *p* = 100 μm, and o = 10 μm

In tissue sections from ≤2-day-old infarcts, IL-1β IR was located in the neuronal cytoplasm, extending out to the proximal dendrites (Fig. 2a) and in glial cells (Fig. 2a, insert) in both I/PI and NAT, whereas IL-1β IR was only located in macrophage-like cells in I/PI.

In tissue sections from 3 to 7-day-old (Fig. 2b) and ≥ 8-day-old (Fig. 2c) infarcts, the cellular distribution of IL-1β IR was comparable to that of ≤2-day-old infarcts. IL-1β IR was observed in glial processes, infiltrating macrophages, intraparenchymal microglia and macrophages, and macrophages located inside blood vessels.

Scoring of the number of IL-1β^+^ cells and their IR yielded the highest score in neurons and glial cells in I/PI and NAT tissue and in macrophages located in I/PI at all timepoints (Fig. 2d). In ≥8-day-old infarcts, the score for IL-1β was highest in I/PI, with no IL-1β^+^ cells in NAT (Fig. 2d, lower graph). Information on IL-1β IR in control tissues is provided in Suppl. Fig. 4.

### IL-1Ra IR is mainly present in glia and infiltrating macrophages

In tissue sections from ≤2-day-old infarcts, IL-1Ra IR was confined to glial cells (Fig. 2e) and neurons in the NAT and I/IP, whereas IL-1Ra IR was located in macrophages only in I/IP. IL-1Ra^+^ macrophages appeared to extravasate from capillaries located in I/IP (Fig. 2e, insert). IL-1Ra IR was located in the cytoplasm and in microglia, also to cellular processes. In tissue sections from 3 to 7-day-old infarcts, IL-1Ra IR was only located in glia in NAT, though still present in neurons, glial cells, and in macrophages in I/PI (Fig. 2f). At ≥8 days, no neuronal IL-1Ra IR was observed, and IL-1Ra IR was only present in glia in NAT and glia and macrophages in I/PI (Fig. 2g).

Scoring of IL-1Ra staining showed that although IL-1Ra IR was present in neurons (Fig. 2h, upper graph), glia scored higher in I/PI and NAT tissue in ≤2-day-old infarcts, and additionally increased in 3–7-day-old and ≥ 8-day-old infarcts (Fig. 2h, middle graph). Finally, scoring of macrophage IL-1Ra staining intensity showed that IL-1Ra^+^ macrophages were only observed in the I/PI, not in NAT (Fig. 2d, lower graph). Information on IL-1Ra IR in control tissues is provided in Suppl. Fig. 5.

### IL-1α IR is present in neurons, glia, and infiltrating macrophages

In tissue sections from ≤2-day-old infarcts, IL-1α IR was located in the cytoplasm of neurons and in glia (Fig. 2i) both in NAT and I/IP. The same was true for 3–7-day-old and ≥ 8-day-old infarcts (Fig. 2j,k), though with decreasing number and IR of both neurons and glia in NAT (Fig. 2l, upper and middle graphs). Scoring showed that the number of IL-1α^+^ macrophages and their IR were low in macrophages in NAT at all timepoints and significantly increased in I/PI tissue from ≤2-day- and 3–7-day-old infarcts (Fig. 2l, lower graph). At ≥8 days, the level of IR was higher in macrophages in I/PI than in NAT, though not statistically significant (Fig. 2l, lower graph). Information on IL-1α IR in control tissues is provided in Suppl. Fig. 6.

### TNF IR is present in ischemic neurons, glia, and infiltrating macrophages

**Fig. 3:**
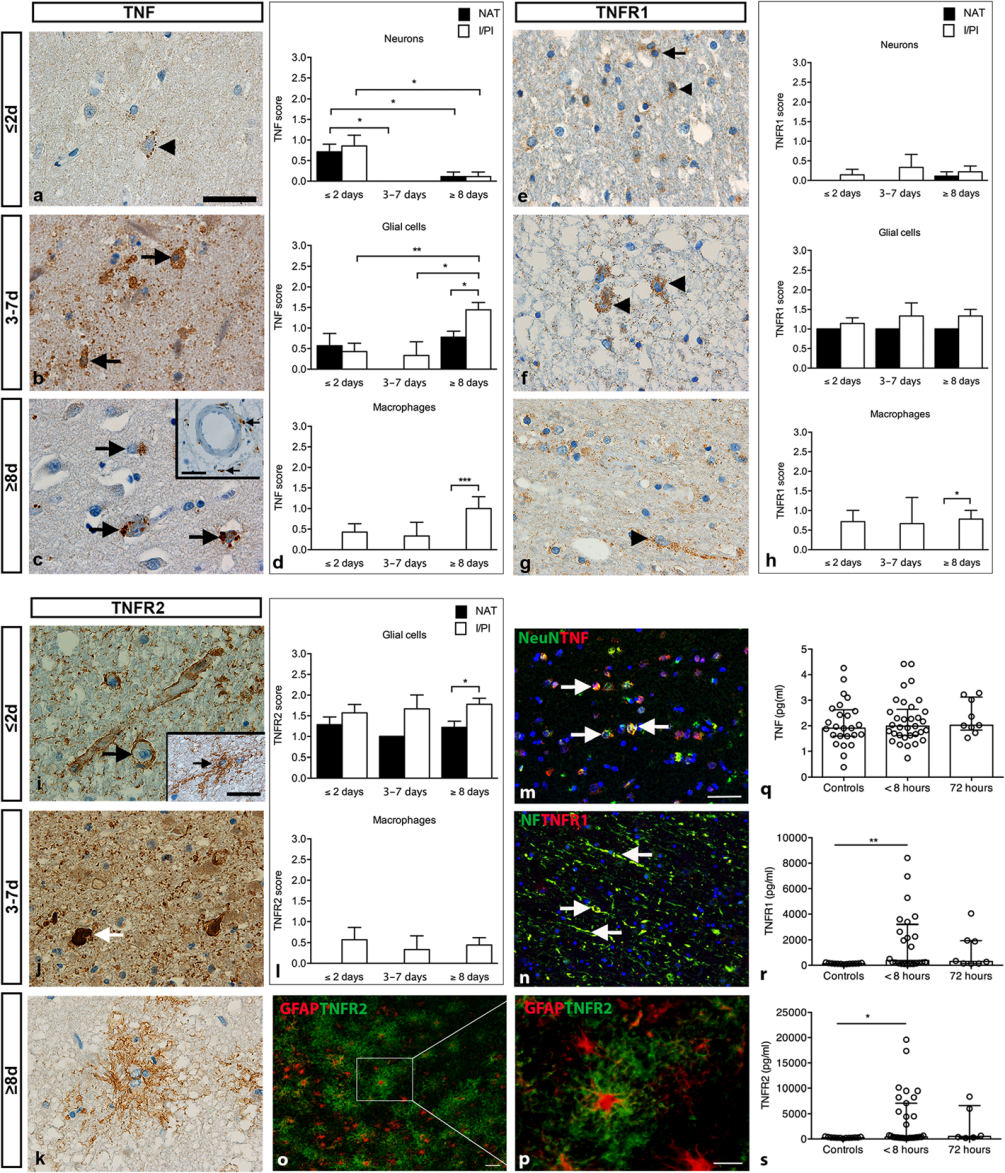
Characterization of cellular localization and plasma levels of TNF, TNFR1, and TNFR2 in ischemic stroke. **a** TNF immunohistochemical staining showing a TNF^+^ neuron (arrowhead) in a ≤ 2-day-old infarct. **b** TNF^+^ glial- and macrophage-like cells (arrows) located in a 3–7-day-old infarct. **c** TNF^+^ cells (arrows) and TNF^+^ macrophages extravasating from capillaries (arrows in insert) in a ≥ 8-day-old infarct. **d** Scoring of TNF staining intensity showing significantly higher TNF expression in neurons located in I/PI in ≤2-day-old infarcts compared to ≥8-day-old infarcts and in neurons located in NAT in ≤2-day-old infarcts compared to both 3–7-day-old and ≥ 8-day-old infarcts (upper graph). TNF expression was comparable in glial cells located in I/PI and NAT in ≤2-day-old and 3–7-day-old infarcts, with significantly increased expression in I/PI in ≥8-day-old infarcts compared to ≤2-day- and 3–7-day-old infarcts and compared to NAT (middle graph). At all timepoints, TNF expression in macrophages was only observed in I/PI (lower graph). **e** TNFR1 immunohistochemical staining demonstrating a TNFR1^+^ glial cell (arrow) and TNFR1^+^ neuron (arrowhead) in a ≤ 2-day-old infarct. **f** TNFR1^+^ neurons (arrowheads) in a 3–7-day-old infarct. **g** TNFR1^+^ neuron (arrowhead) in a ≥ 8-day-old infarct. **h** Scoring of staining intensity showed that TNFR1 expression was comparable in neurons and glia in I/PI and NAT at all timepoints. TNFR1 was mainly expressed in macrophages located in I/PI and absent from NAT. **i** TNFR2 immunohistochemical staining demonstrating TNFR2^+^ glial end-feet encircling the blood vessels (arrow) and a TNFR2^+^ glia cell (insert) in ≤2-day-old infarcts. **j** TNFR2^+^ cell (arrow) in a 3–7-day-old infarct. **k** TNFR2^+^ glia in a ≥ 8-day-old infarct. **l** Scoring of TNFR2 staining intensity showing comparable TNFR2 expression in glia located in I/PI and NAT in ≤2-day-old and 3–7-day-old infarcts and with a significant increase in TNFR2 expression in glia located in I/PI compared to NAT in ≥8-day-old infarcts. TNFR2 expression was only found in macrophages located in I/PI. No neurons were found to express TNFR2. **m** Immunofluorescent staining demonstrating co-localization of TNF (red) with NeuN^+^ (green) neurons (arrows). **n** Immunofluorescent staining demonstrating co-localization of TNFR1 (red) with NF^+^ (green) neurons and their proximal dentrites (arrows). **o** Immunofluorescent staining demonstrating co-localization of TNFR2 (green) with GFAP^+^ astrocytes (red). **p** High magnification of (**o**). **q** V-Plex analysis demonstrating comparable plasma TNF levels between controls and ischemic stroke patients at < 8 and 72 h after symptom onset. **r-s** V-Plex analysis demonstrating significantly increased plasma TNFR1 (r) and TNFR2 (s) levels in ischemic stroke patients < 8 h after symptom onset compared to controls. For TNF, CV > 25% in three control samples and one < 8-h sample. For TNFR1, CV > 25% in four < 8-h samples, 12 control samples, and one 72-h sample. For TNFR2, CV > 25% in three < 8-h samples, 11 control samples, and three 72-h samples. GFAP, glial fibrillary acidic protein; I/PI, infarct/peri-infarct; NF, neurofilament; NAT, normal appearing tissue; TNF, tumor necrosis factor; TNFR1, tumor necrosis factor receptor 1; TNFR2, tumor necrosis factor receptor 2. Results are presented as mean ± SD (staining intensity) or mean with IQR (V-plex analysis). *p < 0.05, **p < 0.01, ***p < 0.001; one-way ANOVA followed by Sidak’s multiple comparisons test (scoring of staining intensity, *n* = 3–9/group) or Kruskal Wallis test followed by Dunn’s multiple comparison test (V-plex analysis). Scale bars: a-c, e-g, i-k, p, inserts = 40 μm, and m-o = 100 μm

In tissue sections from ≤2-day-old infarcts, TNF IR was located to small vesicles in the cytoplasm or in association with the cellular surface of neurons (Fig. 3a), glia, and macrophages. In 3–7-day-old infarcts, TNF IR was mainly located to glia and macrophages located within the infarct (Fig. 3b). At ≥8 days, TNF^+^ cells were primarily macrophages located in I/PI and glia located in I/PI and NAT (Fig. 3c), however, neuronal TNF IR was also observed. TNF IR was both observed in glial processes, infiltrating macrophages, and macrophages within blood vessels.

Scoring of TNF staining intensity showed that the number of TNF^+^ cells and their IR were highest in I/PI and NAT neurons in ≤2-day-old infarcts, with decreasing score in tissue sections from patients with 3–7-day-old and ≥ 8-day-old infarcts (Fig. 3d, upper graph). Scoring of TNF staining in glia showed a significantly increased TNF score in I/PI in ≥8-day-old infarcts compared to ≤2-day-old and 3–7-day-old infarcts (Fig. 3d, middle graph). Glial TNF IR was also observed in NAT (Fig. 3d, middle graph). Scoring of macrophage TNF staining yielded a high TNF score in I/PI, with no TNF IR in NAT (Fig. 3d, lower graph). Information on TNF IR in control tissues is provided in Suppl. Fig. 7.

### TNFR1 IR is primarily in neurons but is also upregulated in macrophages and glia

In general, TNFR1 IR was mainly located to neurons, extending into their proximal dendrites (Fig. 3e,g). At ≤2 days, TNFR1 IR was primarily located to neurons and their proximal dendrites (Fig. 3e) but also to glial cells and macrophages (insert in Fig. 3e). At 3–7 days, TNFR1 IR was still observed in neurons but also in infiltrating macrophages and glia (Fig. 3f). At ≥8 days, TNFR1 IR was primarily observed in neurons (Fig. 3g), but a few macrophages and glia also upregulated their TNFR1 IR. Scoring of TNFR1 staining in neurons showed that TNFR1 IR was mainly found in I/PI tissue at all timepoints (Fig. 3h, upper graph). Glia located in both I/PI and NAT showed TNFR1 IR (Fig. 3 h, middle graph), and macrophages in I/PI tissue showed TNFR1 IR (Fig. 3h, lower graph). Information on TNFR1 IR in control tissues is provided in Suppl. Fig. 8.

### TNFR2 IR is primarily in astrocytes, but also in microglia and macrophages

Astrocytes (Fig. 3i) were the most predominant cell type showing TNFR2 IR in ≤2-day-old infarcts. At 3–7 days, also macrophages in I/PI tissue (Fig. 3j) in addition to astrocytes showed TNFR2 IR. At ≥8 days, TNFR2 IR was also observed in glia (Fig. 3k). At all timepoints, TNFR2 IR was associated to the cellular processes. Scoring of TNFR2 staining intensity demonstrated that glia in I/PI and NAT showed TNFR2 IR at all timepoints (Fig. 3l, upper graph). In addition, TNFR2 IR was located to macrophages in the I/PI area at all timepoints (Fig. 3l, lower graph). No neuronal TNFR2 IR was observed. Information on TNFR2 IR in control tissues is provided in Suppl. Fig. 9.

### IL-1α, IL-1β, IL-1Ra, and TNF IR is located to subsets of cells

**Fig. 4:**
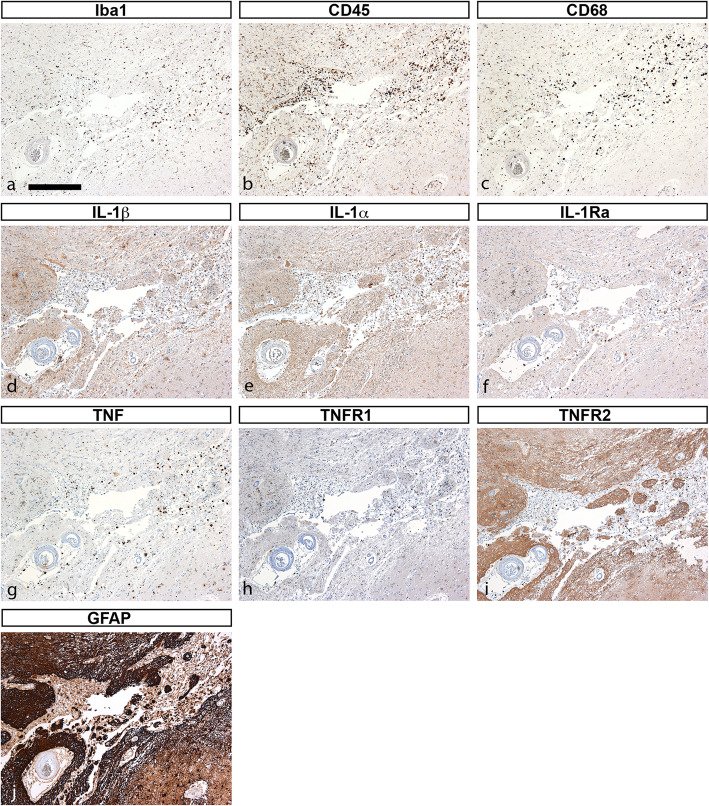
Characterization of glial markers, IL-1, TNF, and TNF receptors in post-mortem tissue sections. **a-j** Immunohistochemical staining of parallel tissue sections from a < 1-day-old infarct for glial markers Iba1 (**a**), CD45 (**b**), and CD68 (**c**), and for cytokines IL-1β (**d**), IL-1α (**e**), IL-1Ra (**f**), and TNF (**g**), and for TNFR1 (**h**), TNFR2 (**i**), and the astrocyte marker GFAP (**j**). GFAP, glial fibrillary acidic protein; IL-1α, interleukin-1alpha; IL-1β, interleukin-1beta; IL-1Ra, interleukin-1 receptor antagonist; TNF, tumor necrosis factor; TNFR1, tumor necrosis factor receptor 1; TNFR2, tumor necrosis factor receptor 2. Scale bar = 40 μm

**Fig. 5:**
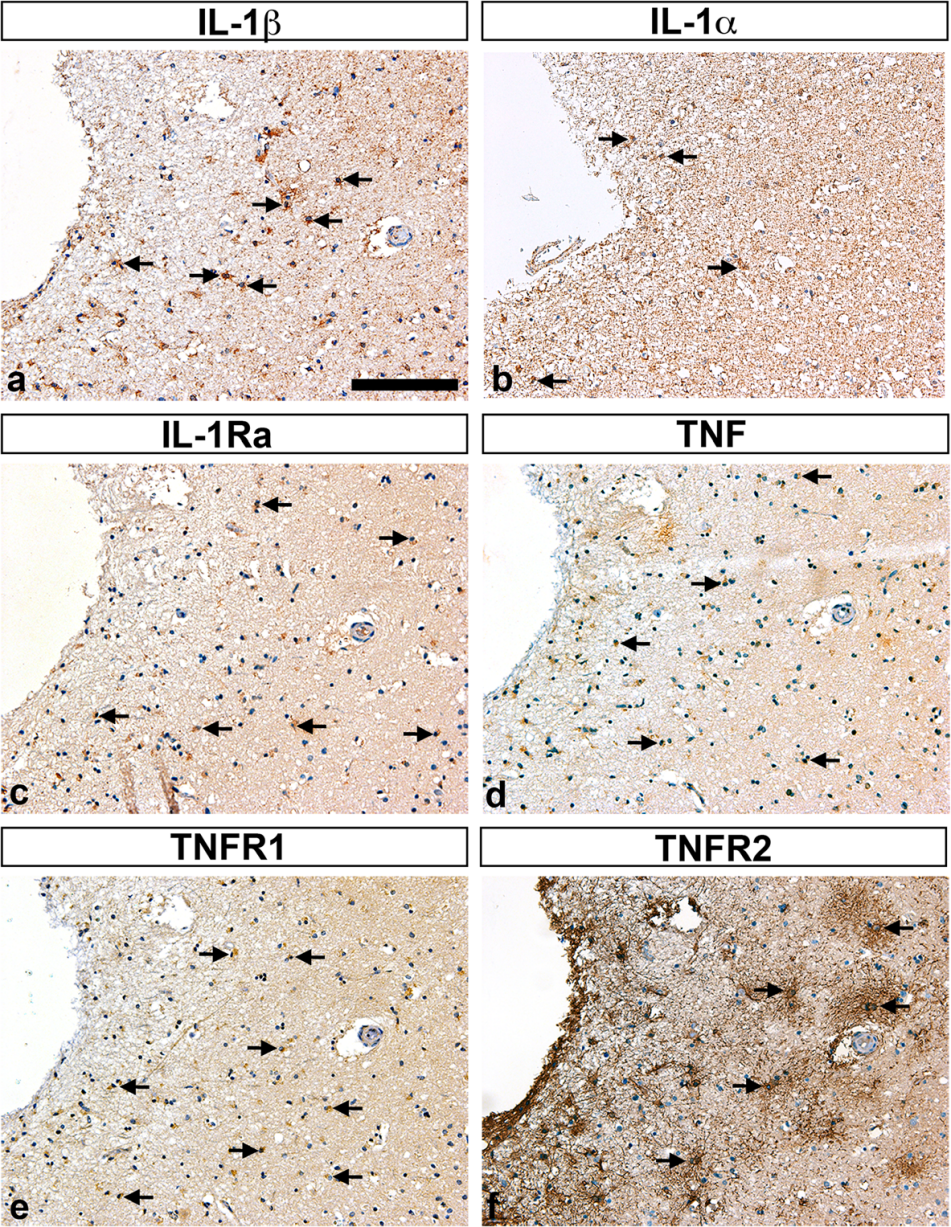
Characterization of IL-1, TNF, and TNF receptors TNFR1, and TNFR2 in post-mortem tissue sections. **a-f** Immunohistochemical staining of parallel tissue sections from a > 7-day-old infarct for cytokines IL-1β (arrows in **a**), IL-1α (arrows in **b**), IL-1Ra (arrows in **c**), and TNF (arrows in **d**), and for TNFR1 (arrows in **e**), and TNFR2 (arrows in **f**). IL-1α, interleukin-1alpha; IL-1β, interleukin-1beta; IL-1Ra, interleukin-1 receptor antagonist; TNF, tumor necrosis factor; TNFR1, tumor necrosis factor receptor 1; TNFR2, tumor necrosis factor receptor 2. Scale bar = 40 μm

We showed previously that TNF and IL-1β [11] and IL-1α, IL-1β, IL-1Ra [12] are produced by subsets of microglia in experimental stroke. We therefore compared parallel tissue sections from two stroke cases stained for IL-1α, IL-1β, IL-1Ra, TNF, TNFR1, and TNFR2 (Figs. 4 and 5). The first (case #7, Table 1) was a <  1-day-old right frontal lobe infarct in an 80-year-old female (Fig. 4). In line with our findings for all infarcts ≤2-day-old, we observed increased Iba1 (Fig. 4a), CD45 (Fig. 4b), and CD68 (Fig. 4c) IR in microglia/macrophages located within the infarct. Similar to our experimental stroke studies, IL-1β (Fig. 4d), IL-1α (Fig. 4c), IL-1Ra (Fig. 4f), and TNF (Fig. 4g) IR was located to subsets of cells (please compare Fig. 4d-g). TNFR1 IR was sparse (Fig. 4h), and TNFR2 IR (Fig. 4i) appeared to co-localize with areas of increased GFAP IR (Fig. 4j), suggesting that TNFR2 is expressed primarily by astrocytes. The second case (case #9, Table 1) was a >  7-day-old caudate nucleus infarct in a 68-year-old male (Fig. 5). Comparing parallel tissue sections, we observed that at this timepoint, IL-1β (Fig. 5a), IL-1α (Fig. 5b), IL-Ra (Fig. 5c), and TNF (Fig. 5d) IR was located to subsets of cells within the infarct. TNFR1 IR appeared to be increased on microglial-like cells and macrophages located within the infarct (Fig. 5e), whereas TNFR2 IR appeared to be increased on astrocyte-like cells (Fig. 5f).

These studies prompted us to perform co-localization studies, trying to identify the cellular sources of IL-1α, IL-1β, IL-1Ra (Fig. 2m-0), TNF, TNFR1, and TNFR2 (Fig. 3m-p). We found that IL-1β co-localized both to Iba1^+^ microglia (Fig. 2m), Iba1^+^ macrophage-like cells (insert in Fig. 2 m), and NF^+^ neurons (Fig. 2n). IL-1Ra IR co-localized to CD45^+^ cells (Fig. 2o), and IL-1α to Iba1^+^ (Fig. 2p) and CD68^+^ (insert in Fig. 2p) cells. TNF IR co-localized to NeuN^+^ neurons (Fig. 3m) and CD45^+^ cells (not shown), and TNFR1 to NF^+^ neurons and their proximal dendrites (Fig. 3n), while TNFR2 IR co-localized to GFAP^+^ astrocytes (Fig. 3o-p). The double immunofluorescent staining experiments thereby support the immunohistochemical staining and the qualitative scores.

### Serum IL-1α, IL-1β, and IL-1Ra levels do not change in ischemic stroke patients

Baseline characteristics of ischemic stroke patients and controls are presented in Table 2. Importantly, we observed no difference in the use of anti-inflammatory medicine (*p* = 0.21) between ischemic stroke patients and controls.

**Table 2:** Characteristics of study participants

Variable	Controls	Ischemic stroke	*p*-value
Men, n (%)	19 (68)	19 (56)	0.34
Age, years (mean ± SD)	59.3 ± 12.8	64.5 ± 12.8	*0.03
BMI, kg/m^2^ (mean ± SD)	26.1 ± 3.8	26.6 ± 5.5	0.84
SSS, points (mean ± SD)		49.5 ± 9.2	
mRS, points (mean ± SD)		1.8 ± 1.8	
Smoking			**0.003
- Current smokers, n (%)	4 (14.2)	9 (26.4)	
- Previous smokers, n (%)	5 (17.9)	17 (50.0)	
- Non-smokers, n (%)	14 (50)	4 (11.8)	
- Not known, n (%)	5 (17.9)	4 (11.8)	
Alcohol consumption ^a^			*0.04
< health authorities’ recommendations, n (%)	18 (64.3)	25 (73.5)	
> health authorities’ recommendations, n (%)	2 (7.1)	9 (26.5)	
Not known, n (%)	8 (28.6)		
Anti-inflammatory medication, n (%)	4 (14.2)	14 (41.2)	0.21
- Topical steroids	1 (3.6)	8 (23.5)	
- NSAID	3 (10.7)	5 (14.7)	
- Systemic glucocorticoid	3 (10.7)	2 (5.9)	
- Antibodies		1 (2.9)	
Arterial supply area affected			
- Anterior		16 (52.9)	
- Posterior (PCA & basilar/vertebral)		6 (17.7)	
- Nuclei		5 (14.7)	
- Brain stem & spinal cord		3 (8.8)	
- Fiber tracts		3 (8.8)	
- Not identified		1 (2.9)	
Treatment			
- Thrombolysis		5 (14.7)	
- Thrombectomy		5 (14.7)	
Time to first sample, hours (mean ± SD)		7.5 (5.6)	

*BMI* body mass index, *mRS* modified Rankin Scale, *NSAID* non-steroidal anti-inflammatory drug, *PCA* posterior communicating artery, *SSS* Scandinavian Stroke Scale

^a^The Danish Health authority recommends < 7 units per week for women (1 unit equals 1 glass of wine) and < 14 units per week for men

Serum IL-1β was almost undetectable, and we observed no significant difference in IL-1β levels between controls (< 0.000001 pg/ml [< 0.000001;< 0.000001]) and ischemic stroke patients when serum was sampled either < 8 h (< 0.000001 pg/ml [< 0.000001;0.00016]) or 72 h after the vascular event (0.0013 pg/ml [< 0.000001;0.005]) (*p* = 0.47) (Fig. 2q).

No significant differences were observed in serum IL-1Ra levels between controls (237.7 pg/ml [232.2;320.6]) and ischemic stroke patients when serum was sampled either < 8 h (234.2 pg/ml [192.9;336.3]) or 72 h after the vascular event (202.6 pg/ml [169.2;239.2]) (*p* = 0.90) (Fig. 2r).

Finally, IL-1α was only detected in two ischemic stroke patients (Fig. 2s) and thus did not differ between groups.

Similar findings were obtained when analyzing plasma samples for IL-1α, IL-1β, and IL-1Ra (data not shown).

### Plasma TNFR1 and TNFR2 are upregulated in the acute phase after ischemic stroke

We observed no significant differences in plasma TNF levels between controls (1.9 pg/ml [1.6;2.6]) and ischemic stroke patients when plasma was sampled < 8 h (2.0 pg/ml [0.7;2.6]) or 72 h (2.0 pg/ml [1.8;3.1], *n* = 9) after the vascular event (*p* = 0.57) (Fig. 3q).

In contrast, TNFR1 plasma levels were significantly upregulated within 8 h of the vascular event in ischemic stroke patients (376.4 pg/ml [146.8;3202]) compared to controls (131.9 pg/ml [118.5;158.7]) (**p = 0.006) (Fig. 3r). At 72 h after ischemic stroke, TNFR1 levels were not significantly changed (303.3 pg/ml [141.;1936]) compared to controls (Fig. 3r).

Similarly, TNFR2 levels were significantly upregulated within 8 h of the vascular event in ischemic stroke patients (413.8 pg/ml [251.3;7090]) compared to controls (276.7 pg/ml [236.7;357.8]) (*p = 0.04) (Fig. 3s). Although TNFR2 levels were higher at 72 h (518.7 pg/ml [266.7;6605]) compared to < 8 h, we observed no significant difference at this timepoint.

## Discussion

To our knowledge, this study determines for the first time the detailed cellular localization of IL-1α, IL-1β, and IL-1Ra IR in human ischemic stroke. Furthermore, the study adds to the scarce literature on the cellular localization of TNF, TNFR1, and TNFR2 IR. We immunohistochemically characterized neuronal, glial and leukocyte reactions in 14 cases of human post-mortem ischemic stroke, using 21 specimens of infarcts aged 1 to > 8 days. At all timepoints, IL-1α and IL-1β IR was located to neurons and glia in I/PI and NAT, and to macrophages in I/PI. IL-1Ra IR was primarily located to glia in I/PI and NAT, and to macrophages in I/PI. TNF IR was located to neurons in I/PI and NAT ≤2 days after stroke onset, to glia in I/PI and NAT with increased intensity in ≥8-day-old infarcts, and to macrophages in I/PI at all timepoints. TNFR1 IR was located to neurons and glia in I/PI and NAT and to macrophages in I/PI, whereas TNFR2 IR was observed only in glia in I/PI and NAT, and in macrophages in I/PI, being absent in neurons.

The distribution of IL-1α, IL-1β, IL-1Ra, and TNF in parallel sections suggest that these cytokines are expressed by subsets of cells in I/IP and NAT and that TNFR2 is expressed in areas with increased GFAP IR. Supporting this, immunofluorescent double labeling demonstrated that TNFR2 IR co-localized to GFAP^+^ astrocytes and TNFR1 IR to NF^+^ neurons. TNF IR was found in NeuN^+^ neurons and CD45^+^ cells. Furthermore, IL-1α IR co-localized to Iba1^+^ and CD68^+^ cells, IL-1β IR to Iba1^+^ cells and neurons, and IL-1Ra IR to CD45^+^ cells. Finally, plasma TNFR1 and TNFR2 levels increased significantly in the acute phase after symptom onset in ischemic stroke patients compared to healthy controls whereas TNF, IL-1α, IL-1β, and IL-1Ra did not change.

To our knowledge, no other papers besides those published by our laboratory [12, 31] have described the cellular localization of IL-1α, IL-1β, or IL-1Ra in the human ischemic brain. From animal studies we know that IL-1α is primarily expressed by CD11b^+^ microglia and CD41^+^ platelets following focal cerebral ischemia [12, 16]. IL-1β is expressed primarily by CD11b^+^ microglia and macrophages in the acute phase [12, 15] and by astrocytes at more chronic phases [68]. IL-1Ra is preferentially produced by resident microglia [12]. In the present study, we also observed IL-1α, IL-1β, and IL-1Ra IR in glial cells and infiltrating macrophages, however, but we observed IL-1α and IL-1β IR in neurons as well.

Few studies have investigated cellular TNF expression in human post-mortem ischemic brain tissue. Sairanen et al. [51] found TNF to be expressed in neurons 1–6 days post-stroke, peaking at days 2–3, and gone by day 6. Microglial TNF expression was apparent in the acute phase, astrocytic TNF expression dominated later (17–18 days), and leukocytes expressed TNF around day 3. In contrast, two other studies [17, 61] found the most intense TNF IR in microglia and concluded that these cells were the major source. Also in animal models, TNF is primarily expressed by microglia and infiltrating macrophages, although neurons have been reported to express TNF (reviewed in [28]).

Several studies have investigated blood IL-1β levels in ischemic stroke. While some report increased blood IL-1β [55, 62, 69] compared to controls, other studies are in line with our findings and report no changes [18, 20, 48, 59] or even reduced [72] blood IL-1β levels in ischemic stroke patients. Most of these studies were performed in the first 72 h after symptom onset, and the discrepancy in results can be explained by the different timings of blood samples and stroke etiologies. In the study by Tuttolomondo et al. [62] and Licata et al. [34], patients with cardioembolic subtype of stroke had significantly higher plasma IL-1β levels than patients with other stroke subtypes, suggesting that plasma IL-1β changes depend on stroke etiology.

In line with our studies, plasma IL-1α did not change after stroke [72], suggesting that peripheral IL-1α production does not contribute to post-stroke neuroinflammation.

Several studies have demonstrated increased IL-1Ra plasma levels in acute stroke patients [2, 48] and some with an association to functional outcome at 6 months [67]. We observed no significant increase in blood IL-1Ra levels. Given the protective effects of IL-1Ra in experimental animal stroke models (reviewed in [31]), rhIL-1Ra has been administered to ischemic stroke patients within 6 h of symptom onset [19]. Treatment with rhIL-1Ra reduced neutrophil and total white cell counts, C-reactive protein, and IL-6 levels, while 3-month clinical outcomes were better than in placebo-treated. Together with findings of a detrimental role of IL-1β in experimental stroke models, the use of rhIL-1Ra, such as anakinra, holds promise as a neuroprotective treatment in ischemic stroke patients.

In line with our studies, serum TNF levels were unchanged in ischemic stroke patients [18, 20, 48, 69]. Despite the lack of healthy controls, Fassbender et al. [20] found no change in serum TNF levels sampled at 4, 6, 8, 10, and 14 h and 1, 3, 5, and 7 days after symptom onset. Some studies, however, reported on increased blood TNF levels sampled within 12 h [34, 62], 24 h [55, 66, 71], or 72 h [72] after symptom onset in ischemic stroke patients compared to healthy controls, though not in patients with lacunar stroke [34, 62]. In one study, serum TNF levels did not increase until 4–10 days after symptom onset compared to controls [26], suggesting that TNF levels only increase in the chronic phase after ischemic stroke. Adding to the discrepancy in the literature on peripheral TNF levels in ischemic stroke patients, increased plasma TNF correlated with infarct volume in some studies [71] but not in others [26, 55]; with stroke severity at admission in some studies [34, 41, 62] but not in others [72]; and with functional outcome in some studies [41, 71], but not in others [26, 66].

TNFR1 and TNFR2 are associated with arterial stiffness [13, 27] that itself is associated with an increased risk of stroke [39, 65]. One study reported that serum TNFR1 levels predict the risk of recurrent vascular events in patients with lacunar stroke [4]. In another study, high plasma levels of TNFR1 and TNFR2 were associated with incident intracerebral hemorrhage and with poor functional outcome [57], just as increased venous TNFR1 levels were associated with poor outcome in subarachnoid hemorrhage [21]. TNF-mediated inflammation could thus be associated with vascular changes preceding intracerebral hemorrhage. Supporting this, the present study found that plasma TNFR1 and TNFR2 levels increased in acute stroke patients compared to healthy controls. Plasma TNFR1, but not TNFR2, levels were also significantly upregulated in a study [18], where plasma TNFR1 was strongly correlated with CT infarct volume at 5–7 days, and mRS and Barthel Index at 3 and 6 months. Our data differ, however, to findings by Fassbender et al. [20], who found no changes in serum TNFR1 and TNFR2 levels over time in acute ischemic stroke patients. The weakness of this study, however, was the lack of healthy controls. Altogether, this could suggest that the body’s response to ischemic stroke is to increase soluble TNFR1 levels capable of binding soluble TNF, which we know exerts detrimental effects in experimental stroke [70].

In our preclinical stroke model, we have previously shown that IL-1β and TNF are produced by largely segregated populations of microglia and macrophages after experimental stroke in mice [11], providing evidence of the functional heterogeneity among microglia and macrophages post-stroke. This, along with similar findings in the present study, may inform the design of successful anti-inflammatory therapies in stroke. Multiple laboratories have examined IL-1Ra as a therapy in preclinical stroke models in mice (reviewed in [31]). A meta-analysis of the efficacy of IL-1Ra in rodent models of stroke reported on an overall decrease in infarct volumes and an improvement in functional outcomes [42]. In addition, we recently demonstrated, that cell-based delivery of IL-1Ra was neuroprotective and improved functional outcome in mice subjected to ischemia [12] and a multicenter preclinical study examining the short- and long-term effects of subcutaneous IL-1Ra therapy demonstrated consistent decreases in infarct volumes and improvements in neurological deficits for up to 28 days post-treatment [40]. These preclinical data, along with clinical studies demonstrating beneficial effects of IL-1Ra treatment in ischemic stroke patients [19, 54], support IL-1Ra as a candidate therapy for ischemic stroke.

Also, TNF inhibitors hold promise as candidate therapies in ischemic stroke. Peri-spinal administration of the non-selective TNF antagonist etanercept provided benefits for post-stroke pain and improved cognitive dysfunction in chronic stroke patients [50, 60], demonstrating the potential of anti-TNF inhibitors as anti-inflammatory treatment in ischemic stroke. A major barrier to the efficacy of etanercept as a TNF inhibitor in ischemic stroke, however, is its poor blood-brain barrier (BBB) permeability due to its large size. Furthermore, because etanercept targets both solTNF and mTNF, this can predispose patients to an increased risk of systemic infections and demyelinating and cardiovascular diseases [53]. Therefore, formulations of TNF inhibitors with better BBB penetrance and selective inhibition of solTNF have been designed and tested in preclinical stroke models (reviewed in [31]). The use of intravenous injection of cTfRMAb-TNFR, a drug designed to ferry the TNFR2 fusion protein [56] across the BBB using the transferrin receptor (TfR), resulted in reduced infarct volumes and reduced neural deficits 1 and 7 days post-stroke, whereas etanercept had no effect [56]. We previously examined the effect of a selective solTNF inhibitor, XPro1595, versus etanercept in our preclinical stroke model [9, 70]. When XPro1595 and etanercept were given systemically, both improved functional outcome and decreased the acute phase response in the liver, indicating an important effect of tmTNF on the peripheral immune response; however, neither reduced infarct volumes [9]. When XPro1595 or etanercept were delivered directly to the infarcted area for three consecutive days, only XPro1595 significantly reduced infarct volumes, modified microglial activation, and the post-stroke inflammatory response [70], suggesting that targeting solTNF alone may be efficient for the treatment of post-stroke inflammation. Even though more preclinical studies are needed, the use of selective solTNF inhibitors, sparing mTNF, continue to hold promise in stroke therapy.

A limitation of our study is the lack of estimation of blood IL-1 receptor type I and II levels. Plasma levels of IL-1 receptor type II were previously unchanged in ischemic stroke patients within 12 h and up to 1 year after the stroke [18]. Immunohistochemical characterization of the cellular expression of the IL-1 receptors in the brain would have strengthened this paper. Another limitation is the low number of acute ischemic stroke patients and controls included in plasma analyses of peripheral cytokine and receptor levels.

The strengths of the present study are the use of parallel tissue sections from the same post-mortem ischemic stroke brains and the range of controls for specific staining using absorption controls, serum and isotype controls, combined with control tissue from various organs. The thorough scoring of the staining intensity by two independent raters strengthens the conclusions of this paper, as does the inclusion of double immunofluorescent staining (a very tricky protocol to perform on post-mortem human tissue) to verify cellular localization.

## Conclusions

In conclusion, the present study determines the cellular sources of IL-1α, IL-1β, and IL-1Ra in human post-mortem ischemic stroke tissue, and the cellular sources of TNF, TNFR1, and TNFR2 in parallel tissue sections. The results support findings from animal studies [11, 12] that IL-1α, IL-1β, IL-1Ra, and TNF are produced by subsets of primarily microglia and leukocytes in ischemic stroke tissue. Finally, our findings of increased brain cytokines and plasma TNFR1 and TNFR2 support hypotheses that targeting post-stroke inflammation is a promising add-on therapy in ischemic stroke patients.

## Supplementary information

**Additional file 1: Supplementary Figs. 1–9** and **Supplementary Table 1.**

## Data Availability

All data are hosted at Open Patient data Explorative Network (OPEN; https://open.rsyd.dk/), and requests to access datasets should be directed to klambertsen@health.sdu.dk.

## Abbreviations

BBB: Blood-brain barrier
BMI: Body mass index
DAPI: 4′,6′-diamidine-2′-phenylindole dihydrochloride
GFAP: Glial fibrillary acidic protein
HE: Hematoxylin and eosin
Iba1: Ionized calcium-binding adaptor molecule 1
IL-1: Interleukin-1
IL-1Ra: Interleukin-1 receptor antagonist
IR: Immunoreactivity
I/PI: Infarct/peri-infarct
IQR: Interquartile range
mRS: Modified Rankin Scale
NAT: Normal-appearing tissue
NIHSS: National Institute of Stroke Severity Scale
NF: Neurofilament
OUH: Odense University Hospital
PBS: Phosphate-buffered saline
PBS-T: Phosphate-buffered saline containing 0.25% Triton
rh: Recombinant human
SD: Standard deviation
SSS: Scandinavian Stroke Scale
TBS: Tris-buffered saline
TNF: Tumor necrosis factor
TNFR1: Tumor necrosis factor receptor 1
TNFR2: Tumor necrosis factor receptor 2
