# Supplementary material for: Characterization of the TNF and IL-1 systems in human brain and blood after ischemic stroke
Source: Acta Neuropathol Commun. 2020 Jun 5;8:81. doi: 10.1186/s40478-020-00957-y (PMC7273684; doi:10.1186/s40478-020-00957-y)
Supplement: Supplementary file 1 — Additional file 1: Supplementary Figs. 1–9 and Supplementary Table 1. [file 40478_2020_957_MOESM1_ESM.docx]

**Supplemental online material:**

**Characterization of the TNF and IL-1 systems in human brain and blood after ischemic stroke**

**^1,2^Bettina H. Clausen**, ^1^Department of Neurobiology Research, Institute of Molecular Medicine, University of Southern Denmark, J.B. Winsloewsvej 21, st, DK-5000 Odense C, Denmark; ^2^BRIDGE, Inter-Disciplinary Guided Excellence, Department of Clinical Research, University of Southern Denmark, Odense, Denmark, [bclausen@health.sdu.dk](mailto:bclausen@health.sdu.dk)

**^2,3^Martin Wirenfeldt**, ^2^BRIDGE, Inter-Disciplinary Guided Excellence, Department of Clinical Research, University of Southern Denmark, Odense, Denmark; ^3^Department of Pathology, Odense University Hospital, Odense, J.B. Winsloewsvej 15, DK-5000 Odense C, Denmark, [martin.nielsen@rsyd.dk](mailto:martin.nielsen@rsyd.dk)

**^4^Sofie S. Høgedal**, ^4^Department of Neurology, Odense University Hospital, J.B. Winsloewsvej 4, DK-5000 Odense C, Denmark, [sofie.sander.hogedal@rsyd.dk](mailto:sofie.sander.hogedal@rsyd.dk)

**^5,6^Lars H. Frich**, ^5^Orthopedic Research Unit, University of Southern Denmark, Odense, Denmark; ^6^OPEN, Open Patient data Explorative Network, Odense University Hospital, Department of Clinical Research, University of Southern Denmark, J.B. Winsloewsvej 9a, DK-5000 Odense, Denmark, [lars.henrik.frich@rsyd.dk](mailto:lars.henrik.frich@rsyd.dk)

**^1,2,4^Helle H. Nielsen**, ^1^Department of Neurobiology Research, Institute of Molecular Medicine, University of Southern Denmark, J.B. Winsloewsvej 21, st, DK-5000 Odense C, Denmark; ^2^BRIDGE, Inter-Disciplinary Guided Excellence, Department of Clinical Research, University of Southern Denmark, Odense, Denmark, ^4^Department of Neurology, Odense University Hospital, J.B. Winsloewsvej 4, DK-5000 Odense C, Denmark; [helle.hvilsted.nielsen@rsyd.dk](mailto:helle.hvilsted.nielsen@rsyd.dk)

**^3^Henrik D. Schrøder**, ^3^Department of Pathology, Odense University Hospital, Odense, J.B. Winsloewsvej 15, DK-5000 Odense C, Denmark, [henrik.daa.schroeder@rsyd.dk](mailto:henrik.daa.schroeder@rsyd.dk)

**^1^Kamilla Østergaard**, ^1^Department of Neurobiology Research, Institute of Molecular Medicine, University of Southern Denmark, J.B. Winsloewsvej 21, st, DK-5000 Odense C, Denmark, [kamillaoe@gmail.com](mailto:kamillaoe@gmail.com)

**^1,2^Bente Finsen**, ^1^Department of Neurobiology Research, Institute of Molecular Medicine, University of Southern Denmark, J.B. Winsloewsvej 21, st, DK-5000 Odense C, Denmark; ^2^BRIDGE, Inter-Disciplinary Guided Excellence, Department of Clinical Research, University of Southern Denmark, Odense, Denmark, [bfinsen@health.sdu.dk](mailto:bfinsen@health.sdu.dk)

**^2,3^Bjarne W. Kristensen**, ^2^BRIDGE, Inter-Disciplinary Guided Excellence, Department of Clinical Research, University of Southern Denmark, Odense, Denmark; ^3^Department of Pathology, Odense University Hospital, Odense, J.B. Winsloewsvej 15, DK-5000 Odense C, Denmark, [bwk@rsyd.dk](mailto:bwk@rsyd.dk)

**Kate L. Lambertsen*^1,2,4,6^**, ^1^Department of Neurobiology Research, Institute of Molecular Medicine, University of Southern Denmark, J.B. Winsloewsvej 21, st, DK-5000 Odense C, Denmark; ^2^BRIDGE, Inter-Disciplinary Guided Excellence, Department of Clinical Research, University of Southern Denmark, Odense, Denmark; ^4^Department of Neurology, Odense University Hospital, J.B. Winsloewsvej 4, DK-5000 Odense C, Denmark; ^6^OPEN, Open Patient data Explorative Network, Odense University Hospital, Department of Clinical Research, University of Southern Denmark, J.B. Winsloewsvej 9a, DK-5000 Odense, Denmark, [klambertsen@health.sdu.dk](mailto:klambertsen@health.sdu.dk)

***Corresponding author:** Kate Lykke Lambertsen, klambertsen@health.sdu.dk, J.B. Winsloewsvej 21 st., DK 5000, Odense, DK; e-mail klambertsen@health.sdu.dk, tel. +4565503806

**Supplemental material**

**
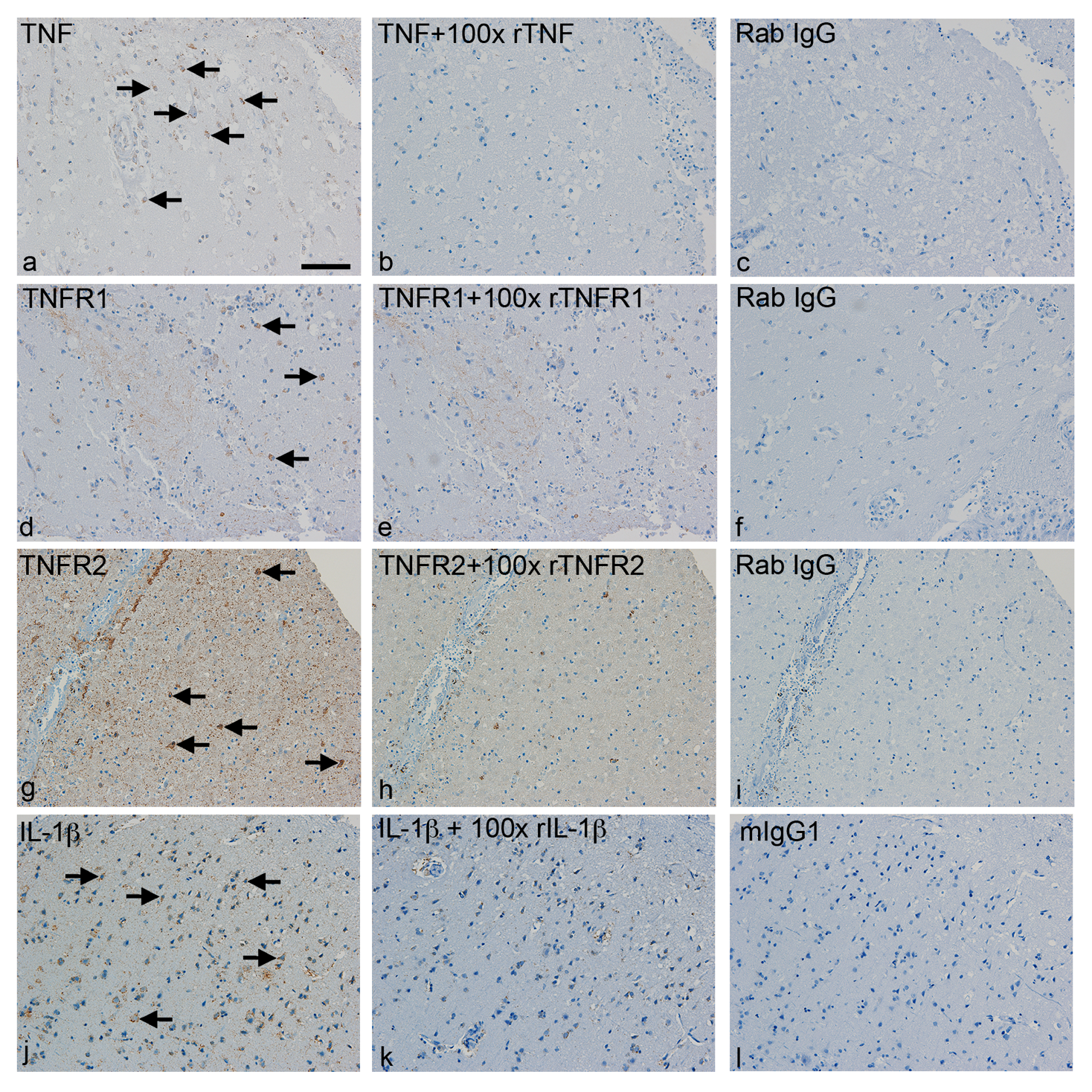
**

**Supplemental Figure 1. Control reactions for immunohistochemical staining experiments.** (**a**) TNF staining demonstrating TNF immunoreactive cells located within the infarcted tissue. (**b**) Absorption control for TNF staining using 100x excess recombinant human TNF demonstrating absence of TNF immunoreactive cells in parallel tissue sections. (**c**) Rabbit IgG serum control for TNF immunohistochemical staining. (**d**) TNFR1 staining demonstrating TNFR1 immunoreactive cells located within the infarcted tissue. (**e**) Absorption control using x100 excess recombinant human TNFR1 for TNFR1 staining demonstrating absence of/decreased TNFR1 immunoreactivity in parallel tissue sections. (**f**) Rabbit IgG serum control for TNFR1 immunohistochemical staining. (**g**) TNFR2 staining demonstrating TNFR2 immunoreactive cells located within the infarcted tissue. (**h**) Absorption control using 100x excess recombinant human TNFR2 for TNFR2 staining demonstrating decreased TNFR2 immunoreactivity in parallel tissue sections. (**i**) Rabbit IgG serum control for TNFR1 immunohistochemical staining. (**j**) IL-1β staining demonstrating IL-1β immunoreactive cells located within the infarcted tissue. (**k**) Absorption control using 100x excess recombinant human IL-1β for IL-1β staining demonstrating absence of IL-1β immunoreactivity in parallel tissue sections. (**l**) Mouse IgG1 isotype control for IL-1β immunohistochemical staining. All images are taken from parallel sections of a 1-day-old right frontal lobe infarct. Scale bar: 100 μm.

**
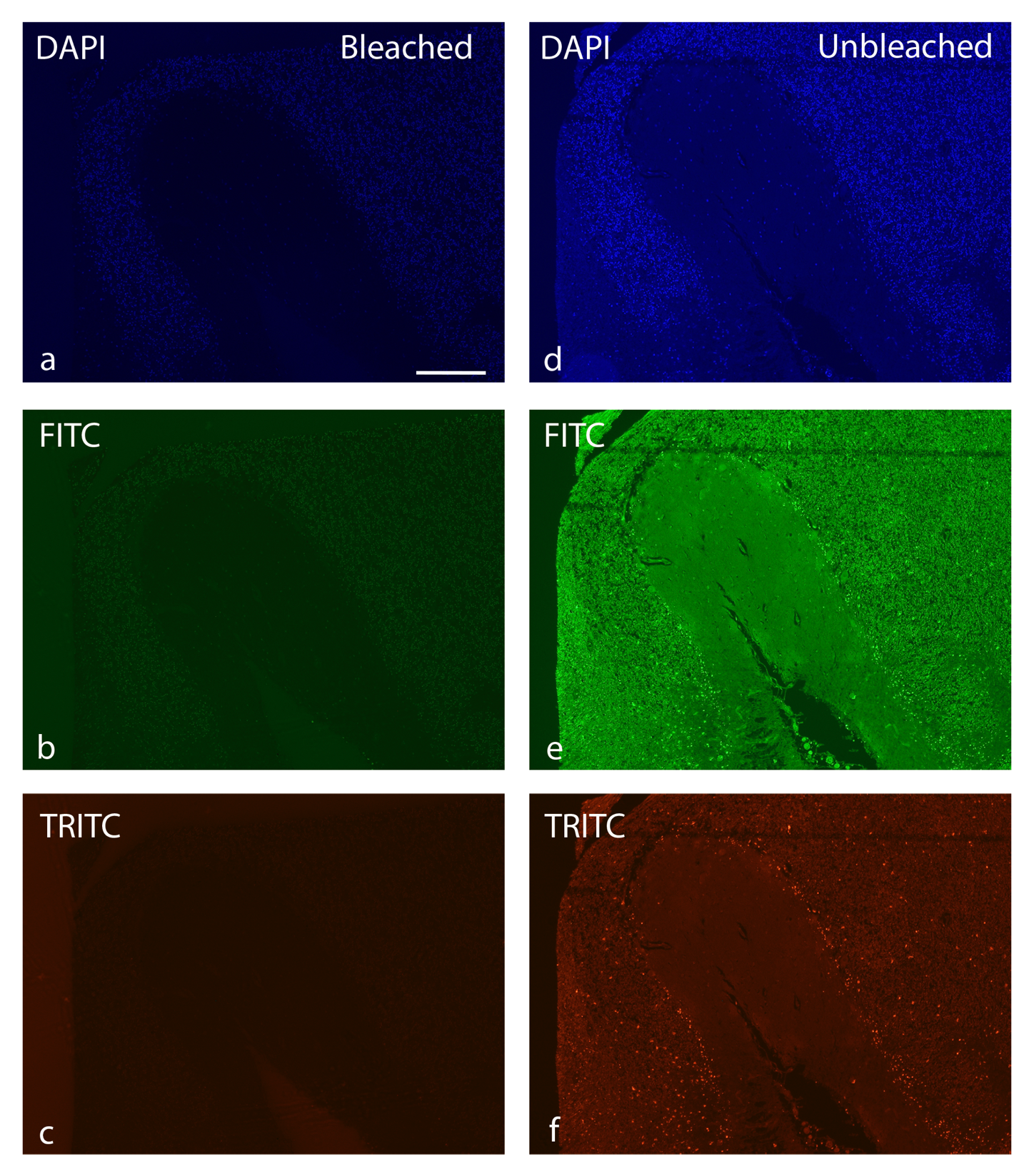
Supplemental Figure 2. Bleaching reduces background autofluorescence.** (**a-c**) Bleaching of tissue sections prior to immunofluorescent staining reduces autofluorescence in both the DAPI (a), FITC (b), and TRITC (c) fluorescence filters. (**d-f**) Autofluorescence from an unbleached tissue section in DAPI (d), FITC (e), and TRITC (f) fluorescence filters. Scale bar: 100 μm.

**
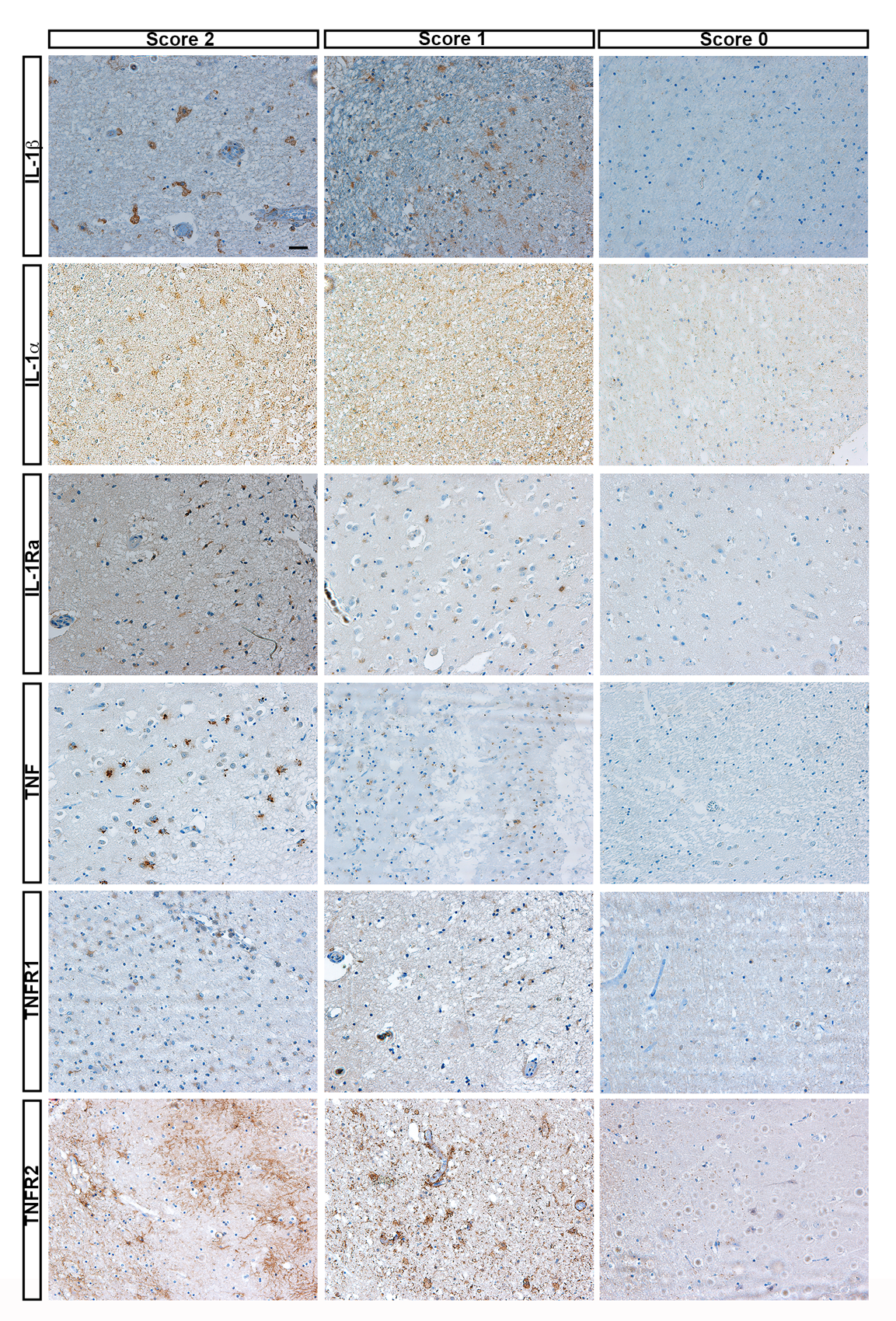
**

**Supplemental Figure 3. Scoring system for immunoreactivity.** Representative images of IL-1β, IL-1α, IL-1Ra, TNF, TNFR1, and TNFR2 stained tissue sections illustrating the scoring system used to score the cell-specific staining intensity. All images were obtained in peri-infarct areas, except for TNFR2 at score 0, which was obtained in normal-appearing tissue, as peri-infarct areas did not score below 1 in TNFR2 stained tissue sections. Scale bar: 50 μm.

**
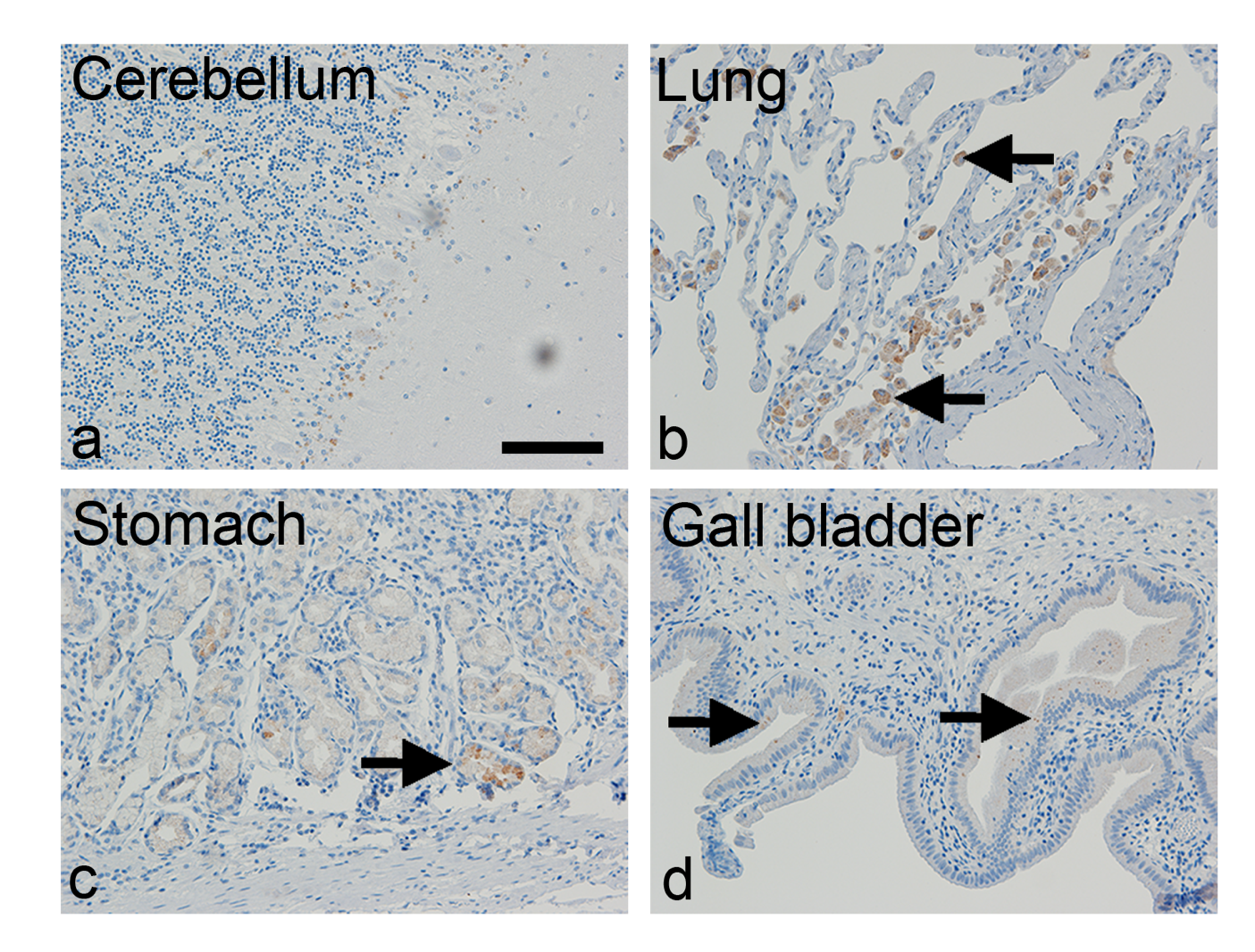
**

**Supplemental Figure 4. Control tissue stained for IL-1β.** (**a-d**) IL-1β expression was found in glial cells of the cerebellum (a), in lung macrophages (arrows in b), in gastric chief cells (arrow in c), and in epithelial cells of the gall bladder (arrows in d). Scale bar: 100 μm.

**
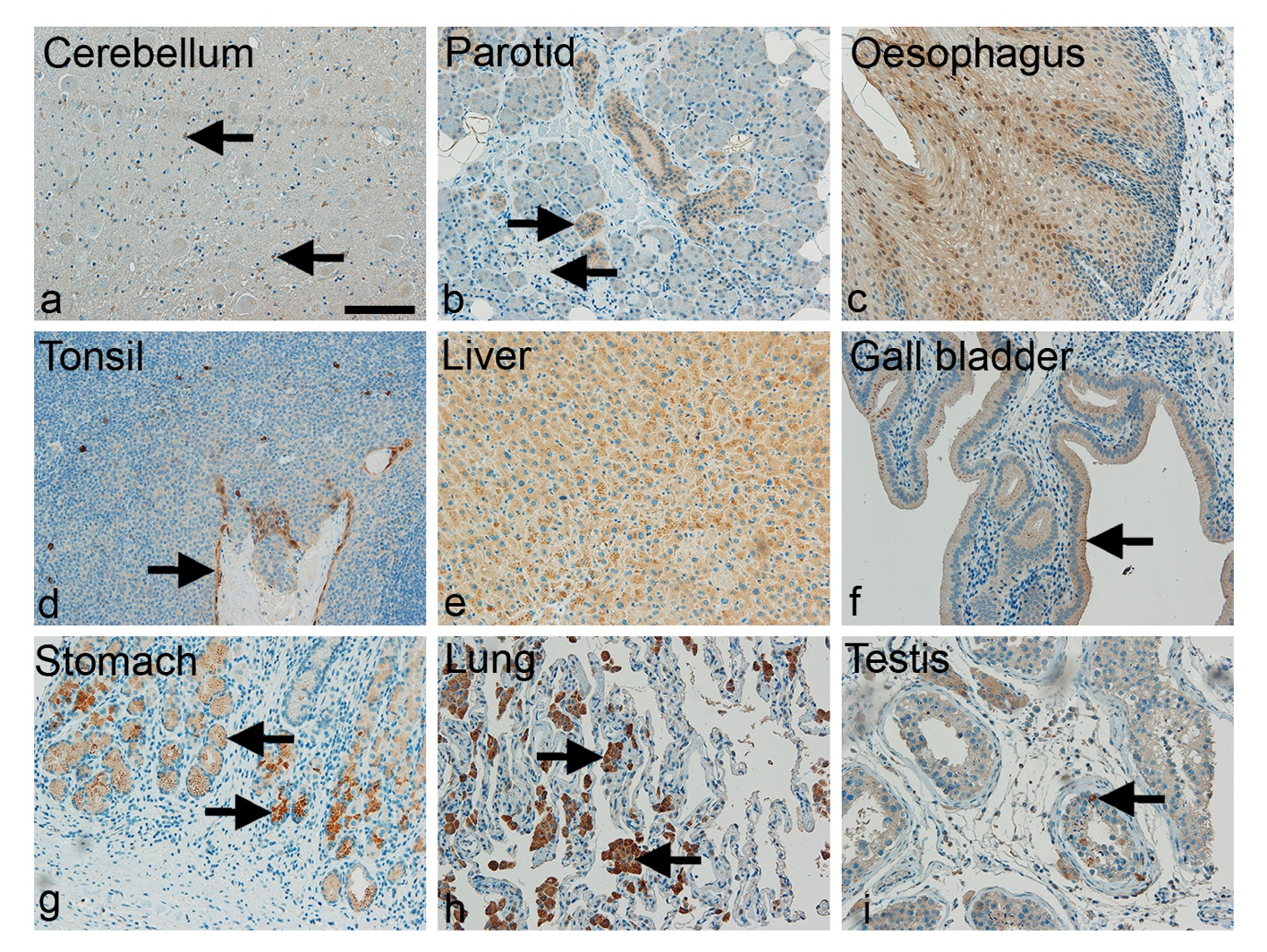
**

**Supplemental Figure 5. Control tissue stained for IL-1Ra.** (**a-i**) IL-1Ra expression was found in glial cells of the cerebellum (arrows in a), in the epithelium of the parotid glandular ducts (arrows in b), in the squamous epithelium and in fibrocytes located in the lamina propria of the oesophagus (c), in the epithelium of the tonsils (arrow in d), in liver hepatocytes (e), in gall bladder epithelium (arrow in f), in gastric chief cells (arrows in g), in lung macrophages (arrows in h), and in Leydig cells of the testis (arrow in g). Scale bar: 100 μm.

**
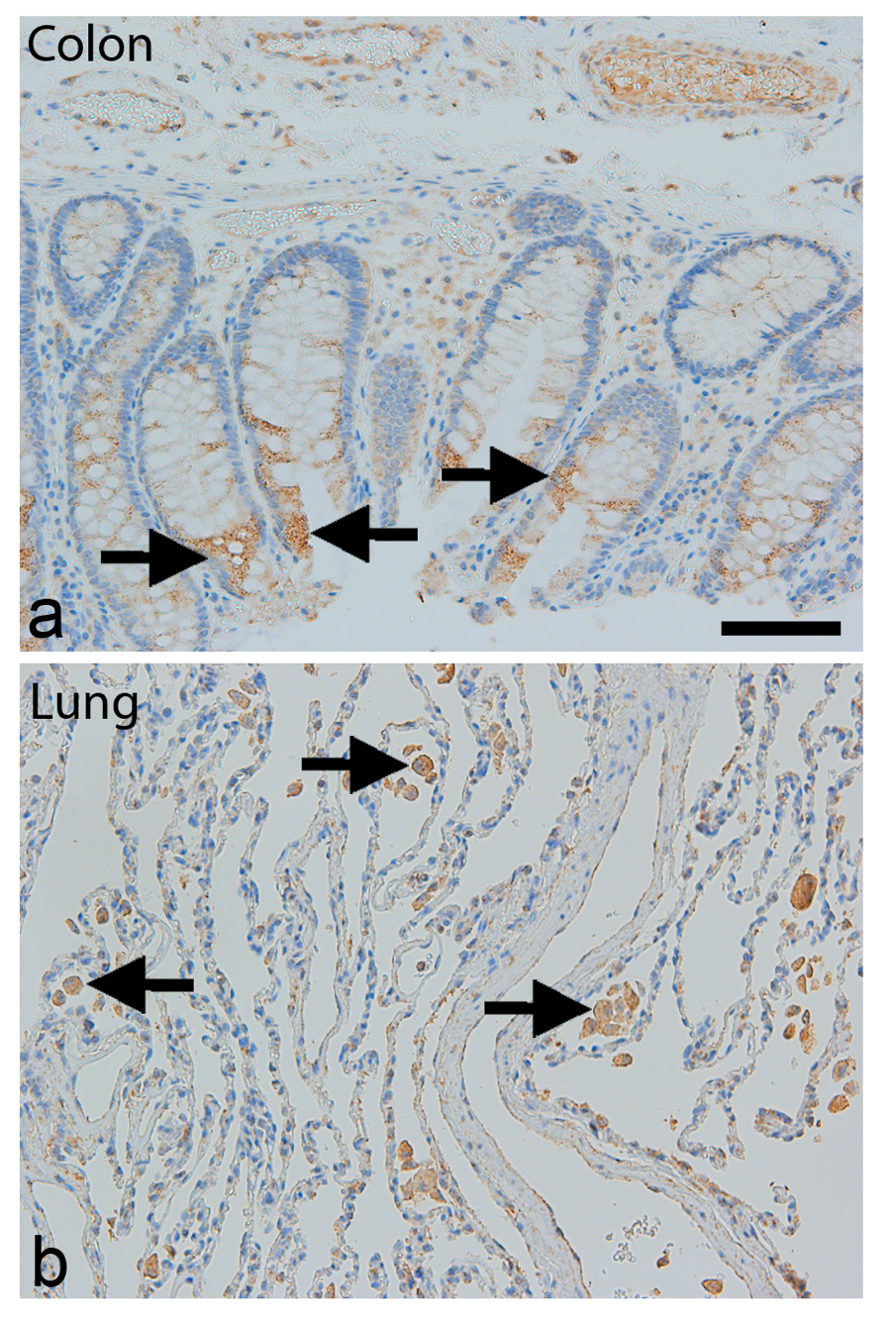
**

**Supplemental Figure 6. Control tissue stained for IL-1α.** (**a-b**) IL-1α expression was found in the cylinder epithelium of the colon (arrows in a) and lung macrophages (arrows in b). Scale bar: 100 μm.

**
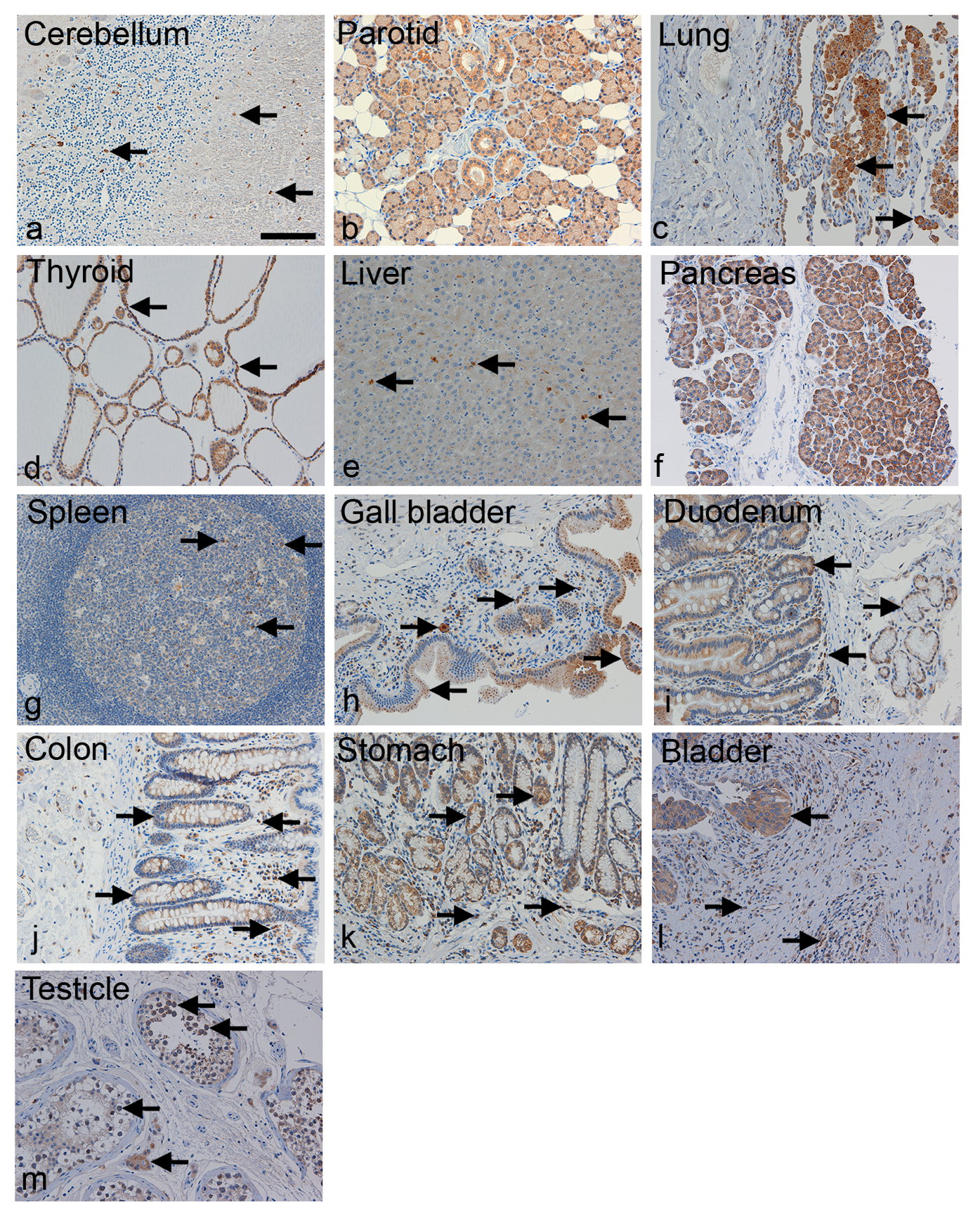
**

**Supplemental Figure 7. Control tissue stained for TNF.** (**a-m**) TNF expression was found in glial cells in the cerebellum (arrows in a), in the epithelium and the end portions of the parotid glandular ducts (b), in lung epithelium and lung macrophages (arrows in c), in thyroid follicle epithelium (arrows in d), in liver Kupffer cells (arrows in e), in pancreatic exocrine gland cells (f), in lymphocytes located in the germinal center of the spleen lymphoid nodules (arrows in g), in gall bladder epithelium and in macrophages located in the lamina propria of the gall bladder (arrows in h), in Paneth cells, Brunner’s glands, and lamina propria of the duodenum (arrows in i), in lamina propria lymphocytes and endothelial cells of the colon (arrows in j), in gastric chief cells and lymphocytes in the stomach (arrows in k), in bladder urothelium and bladder macrophages (arrows in l), and in Leydig and spermatogonia cells of the testis (arrows in m). Scale bar: 100 μm.

**
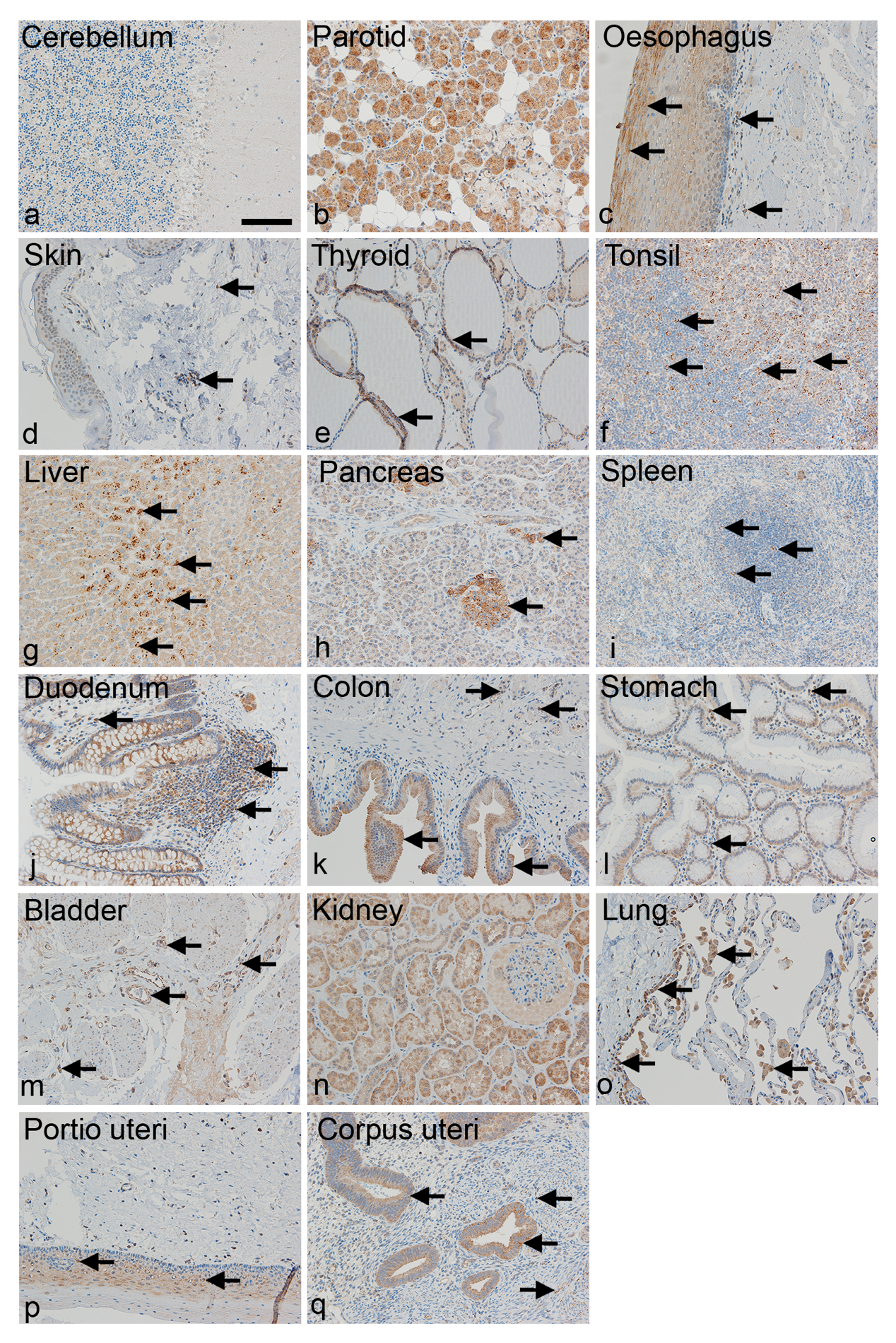
**

**Supplemental Figure 8. Control tissue stained for TNFR1.** (**a-q**) TNFR1 expression was low in the cerebellum (a), but higher in the end portions of the parotid glandular ducts (b), in the squamous epithelium and lymphocytes in the lamina propria of the oesophagus (arrows in c), in lymphocytes located in the skin (arrows in d), in the follicle epithelium of the thyroid (arrows in e), in macrophages in the tonsil (arrows in f), in liver hepatocytes (arrows in g), in the pancreatic islets of Langerhans (arrows in h), in spleen macrophages (arrows in i), in duodenal lymphocytes (arrows in j), in the endothelium and crypt and surface epithelium of the colon (arrows in k), in gastric lymphocytes (arrows in l), and in endothelial cells, lymphocytes and macrophages of the bladder (arrows in m), in the proximal tubules of the kidney (n), in lung epithelium and lung macrophages (arrows in o), in the basal part of the squamous epithelium of the cervix uterus portio (arrows in p), and in the endometrioid glands and lymphocytes of the cervix uterus corpus (arrows in q). Scale bar: 100 μm.

**
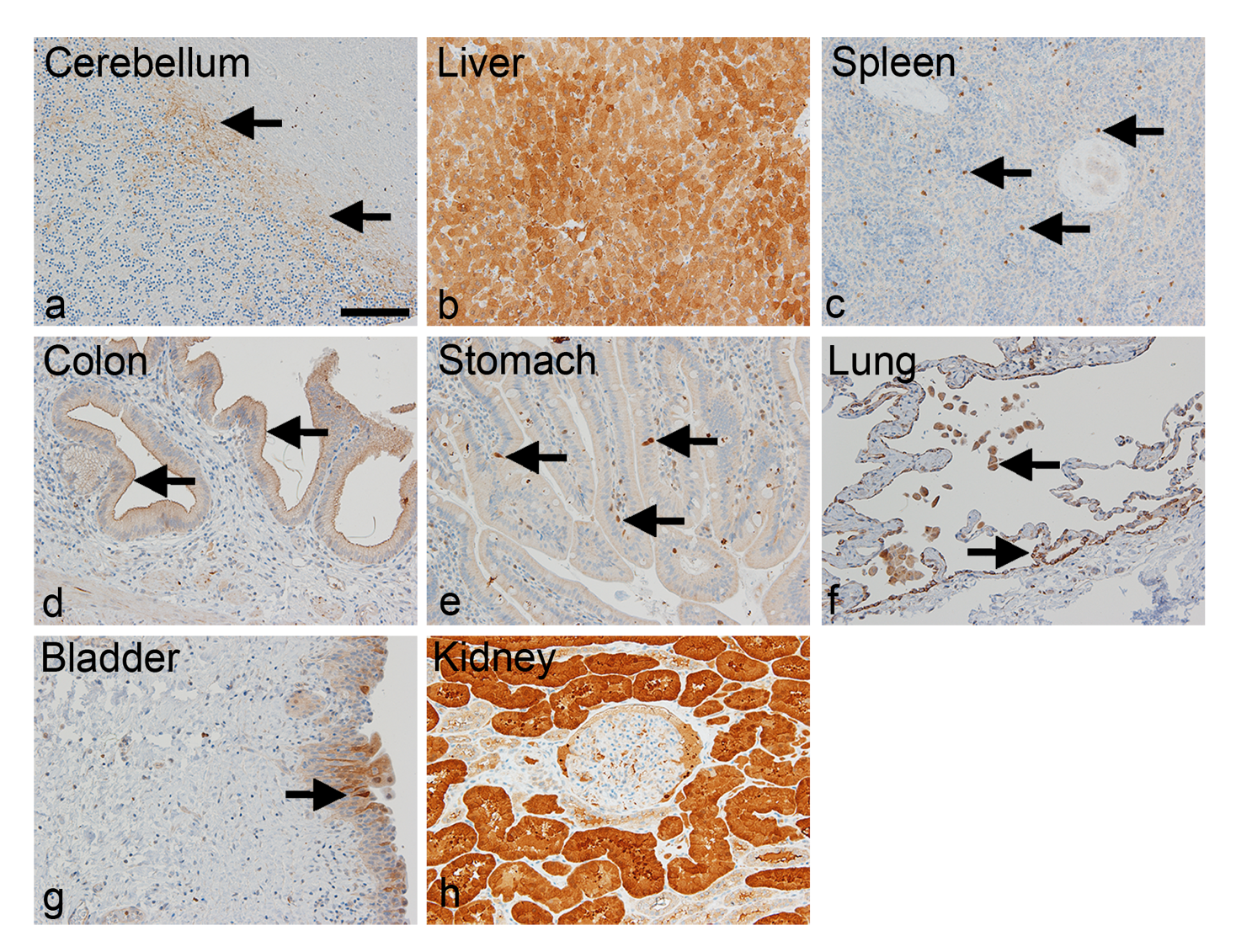
**

**Supplemental Figure 9.** **Control tissue stained for TNFR2.** (**a-h**) TNFR2 expression was found in glial processes in the cerebellum (arrows in a), in liver hepatocytes (b), in macrophages located in the spleen (arrows in c), in the luminal part of colon epithelium (arrows in d), in neuroendocrine cells located in the pyloric part of the stomach (arrows in e), in lung macrophages (arrows in f), in the bladder urothelium (arrow in g), and in the proximal tubules of the kidney (h). Scale bar: 100 μm.

| Variable | Relatives | Orthopedic patients | p-value |
| --- | --- | --- | --- |
| Men, n (%)^a^ | 4 (57) | 15 (71) | 0.65 |
| Age, years (mean ± SD)^b^ | 49.6±19.1 | 62.6±8.16 | 0.09 |
| BMI, kg/m^2^ (mean ± SD)^b^ | 26.6±4.4 | 25.9±3.6 | 0.72 |
| Smoking^c^   - Current smokers, n (%) - Previous smokers, n (%) - Non-smokers, n (%) - Not known, n (%) | 1 (14.3)  4 (57.1)  2 (28.6)  0 (0) | 4 (19.05)  1 (4.8)  12 (57.1)  4 (19.05) | *0.02 |
| Alcohol consumption^c,d^  < health authorities’ recommendations, n (%)  > health authorities’ recommendations, n (%)  Not known, n (%) | 6 (85.7)  1 (14.3)  0 (0) | 12 (57.1)  1 (4.8)  8 (38.1) | 0.14 |
| Anti-inflammatory medication, n (%)^a^ | 1 (14.3) | 4 (19.0) | >0.99 |
| Cytokine and cytokine receptor levels (pg/mL)^b^   - TNF (mean ± SD) - TNFR1 (mean ± SD) - TNFR2 (mean ± SD) | 2.43±1.31  140.8±43.1  303.2±104.6 | 1.98±0.74  134.9±38.7  284.3±112.6 | 0.78  0.95  0.95 |
| Differential leukocyte count^e^ mean±SD (2.5-97.5% PCTL)   - Total leukocyte count (10^9^/L) - Neutrophil count (10^9^/L) - Lymphocyte count (10^9^/L) - Monocyte count (10^9^/L) | (3.5-8.8)  (1.5-7.5)  (1.0-4.0)  (0.2-0.8) | 6.2±1.7 (4.3-9.8)  3.5±1.1 (2.2-6.5)  1.8±0.5 (1.0-2.8)  0.6±0.2 (0.2-1.0) |  |

**Supplemental Table 1. Characteristics of control group.** BMI, body mass index, PCTL, percentile; RC, rotator cuff; TNF, tumor necrosis factor. ^a^ Fisher’s exact test, ^b^Mann-Whitney test, and ^c^Chi-square test. ^d^The Danish Health authority recommends <7 units per week for women (1 unit equals 1 glass of wine) and <14 units per week for men. ^e^Differential leukocyte counts were not available for healthy controls and reference ranges of a normal population are given for comparison
